# Ascorbic Acid Promotes Beneficial Effects on Yield Components and Postharvest Quality of Grafted Guava Under Salt Stress

**DOI:** 10.3390/plants15213279

**Published:** 2026-10-24

**Authors:** Vitor Manoel Bezerra da Silva, Geovani Soares de Lima, Lauriane Almeida dos Anjos Soares, Hans Raj Gheyi, Jackson Silva Nóbrega, Jean Telvio Andrade Ferreira, Flávia de Sousa Almeida, Cassiano Nogueira de Lacerda, Larissa Fernanda Souza Santos, Valeska Karolini Nunes Oliveira, Reynaldo Teodoro de Fátima, Reginaldo Gomes Nobre, Saulo Soares da Silva, Smyth Trotsk de Araújo Silva, Allesson Ramos de Sousa, Weslley Bruno Belo de Souza

**Affiliations:** 1 Academic Unit of Agricultural Engineering, Federal University of Campina Grande, Campina Grande 58430-380, PB, Brazil; vitortn20@gmail.com (V.M.B.d.S.); hans.gheyi@ufcg.edu.br (H.R.G.); jean.telvio@estudante.ufcg.edu.br (J.T.A.F.); cassiano.nogueira@estudante.ufcg.edu.br (C.N.d.L.); larissa.fernanda@estudante.ufcg.edu.br (L.F.S.S.); valeska.karolini@estudante.ufcg.edu.br (V.K.N.O.); smyth.trotsk@estudante.ufcg.edu.br (S.T.d.A.S.); allesson.ramos@estudante.ufcg.edu.br (A.R.d.S.); weslley.bruno@estudante.ufcg.edu.br (W.B.B.d.S.); 2 Academic Unit of Agrarian Sciences, Federal University of Campina Grande, Pombal 58840-000, PB, Brazil; lauriane.almeida@professor.ufcg.edu.br (L.A.d.A.S.); saulo.s.silva@estudante.ufcg.edu.br (S.S.d.S.); 3 Academic Unit of Agronomy, Federal University of Western Pará, Rurópolis 68170-000, PA, Brazil; jackson.nobrega@discente.ufopa.edu.br; 4 Postgraduate Program in Engineering and Management of Natural Resources, Federal University of Campina Grande, Campina Grande 58430-380, PB, Brazil; flavia.almeida@estudante.ufcg.edu.br; 5 Postgraduate Program in Tropical Horticulture, Federal University of Campina Grande, Pombal 58840-000, PB, Brazil; reynaldo.teodoro@estudante.ufcg.edu.br; 6 Department of Science and Technology, Federal Rural University of the Semi-Arid Region, Mossoró 59625-900, RN, Brazil; reginaldo.nobre@ufersa.edu.br

**Keywords:** *Psidium guajava* L., antioxidant, abiotic stress

## Abstract

This study aimed to evaluate the effects of foliar application of ascorbic acid on the yield and postharvest quality of guava fruits under irrigation with saline water during the post-grafting stage. The experiment was conducted under field climatic conditions using lysimeters in a randomized complete block design with a split-plot arrangement. Five irrigation water electrical conductivity levels (ECw: 0.9, 1.5, 2.1, 2.7, and 3.3 dS m^−1^) were assigned to the main plots, while four ascorbic acid concentrations (Asc: 0, 200, 400, and 600 mg L^−1^) were assigned to the subplots, with three replicates. Guava cv. Paluma was used as the scion, and BRS Guaraçá was used as the rootstock. According to the response surface models, foliar application of 600 mg L^−1^ Asc increased fruit diameter and yield performance of ‘Paluma’ guava under 0.9 dS m^−1^, whereas under 3.3 dS m^−1^, it increased average fruit weight and improved quality attributes, including pH, titratable acidity, vitamin C, and total sugars. In addition, salinity increased fruit soluble solids, while the MGIDI identified combinations involving 600 mg L^−1^ Asc as having the best multivariate performance. These results indicate the potential of Asc as a viable strategy for guava cultivation under saline conditions.

## 1. Introduction

Guava (*Psidium guajava* L.) is a fruit crop of considerable socioeconomic importance, with its production widely distributed across tropical regions worldwide [1]. Due to the versatility of its fruits, the crop has broad applications in the food industry, being used in the production of sweets, juices, jams, pulp, and preserves [2]. In addition to its economic importance, guava has high nutritional and functional value, notable for its high levels of vitamin C, antioxidant compounds, and essential minerals, as well as antimicrobial and hypoglycemic properties that contribute to its health benefits [3,4].

Despite the potential of guava cultivation in the Brazilian Northeast, this region faces limitations due to the uneven distribution of rainfall in time and space, coupled with high evaporative demand. These conditions may favor salt concentration and the salinization of water sources, increasing the need to use brackish water for irrigation [5]. However, the use of such water can cause osmotic and ionic imbalances, triggering metabolic and physiological alterations that impair plant growth and development [6,7]. Fruits have a lower transpiration rate than leaves. Nevertheless, under salt stress, plants may prioritize the maintenance of leaves and metabolically active tissues, allocating greater amounts of photoassimilates and water resources to these organs. This reduces the availability of water and nutrients to fruits, thereby compromising the fruit-filling process [8,9,10].

Under greenhouse conditions using drainage lysimeters, irrigation with water having an electrical conductivity greater than 2.15 dS m^−1^ compromises the growth and morphophysiological development of guava plants during the post-grafting stage [11]. However, the severity of salinity-induced damage may vary depending on the duration of exposure, irrigation management, crop developmental stage, and edaphoclimatic conditions [12]. In this regard, the use of different rootstock scion combinations represents a promising approach to enhancing plant salt tolerance, as selecting cultivars with distinct physiological traits may improve adaptation to salinity stress and help maintain plant growth and yield performance under saline conditions [13].

In the literature, studies addressing the effects of salt stress on guava have predominantly focused on plants propagated sexually (from seeds) or by cuttings. In a study evaluating the effects of irrigation water electrical conductivity on guava seedling development, Xavier et al. [14] found that irrigation water with an electrical conductivity above 1.6 dS m^−1^ negatively affected the synthesis of photosynthetic pigments and photosystem II efficiency. Similarly, Lacerda et al. [15] observed that high salt concentrations in irrigation water inhibited growth, gas exchange, and yield components in guava plants propagated by cuttings.

Another management strategy for the use of water with high concentrations of dissolved salts in guava production is the foliar application of ascorbic acid (Asc) [16,17]. Asc is a non-enzymatic compound that directly participates in the plant antioxidant system by modulating the balance between the production of reactive oxygen species (ROS) and the capacity of the antioxidant system to neutralize them [18]. However, under stress conditions, endogenous Asc production may be insufficient to maintain redox homeostasis and control excessive ROS accumulation, resulting in cellular damage that may compromise fruit yield and quality [19]. In addition to its antioxidant function, Asc contributes to maintaining plant water status and may favor the redistribution of water and photoassimilates through the phloem, as well as the regulation of phytohormones, such as gibberellins (GA) and indole-3-acetic acid (IAA), which are important for fruit growth and development [20].

Considering the socioeconomic importance of guava in Brazilian agribusiness, it is necessary to develop effective strategies for managing water salinity, particularly in the Brazilian semiarid Northeast. In this context, this study was based on the hypothesis that foliar application of Asc improves fruit yield and the physical and chemical attributes of guava fruits during the post-grafting stage. Therefore, the objective was to evaluate the effects of foliar application of ascorbic acid on the yield components and postharvest attributes of guava fruits cv. Paluma irrigated with different irrigation water electrical conductivity levels in the Brazilian semiarid Northeast.

## 2. Results

### 2.1. Yield Components

The interaction between irrigation water electrical conductivity (ECw) and ascorbic acid (Asc) significantly affected fruit polar diameter (FPD), fruit equatorial diameter (FED), average fruit weight (AFW), fruit yield (FY), and total number of fruits (TNF) of guava cv. Paluma (Table S1).

**Figure 1:**
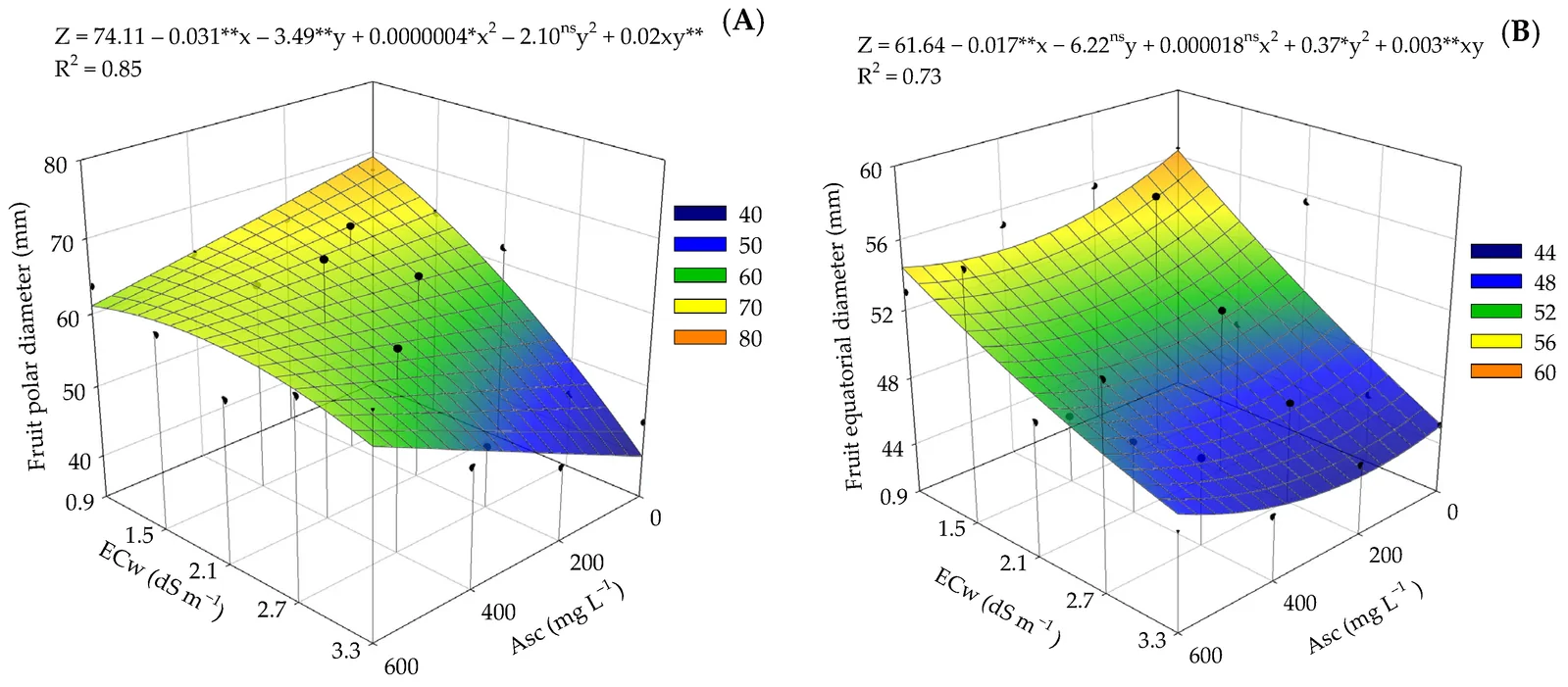
Fruit polar diameter (FPD) (**A**) and fruit equatorial diameter (FED) (**B**) of guava cv. Paluma as affected by the interaction between irrigation water electrical conductivity (ECw) and ascorbic acid concentration (Asc). X and Y represent ascorbic acid concentration (Asc) and irrigation water electrical conductivity (ECw). ^ns^, not significant (*p* > 0.05); *, ** significant at *p* ≤ 0.05 and *p* ≤ 0.01 by the F-test, respectively.

Fruit polar diameter (Figure 1A) of guava plants was negatively affected by the increase in irrigation water electrical conductivity, with the lowest value (39.72 mm) observed at an ECw of 3.3 dS m^−1^ without Asc application, representing a 42.65% reduction in FPD compared with plants irrigated with an ECw of 0.9 dS m^−1^. The highest estimated fruit polar diameter (FPD), at 69.27 mm, was observed in the absence of ascorbic acid (Asc; 0 mg L^−1^) at the lowest irrigation water electrical conductivity (ECw; 0.9 dS m^−1^). In contrast, the beneficial effect of applying ascorbic acid at 600 mg L^−1^ was observed in plants irrigated with water at an ECw of 3.3 dS m^−1^, resulting in a 53.23% increase compared with plants subjected to the same salinity level without Asc application.

For fruit equatorial diameter (Figure 1B), a 19.88% reduction was observed with the increase in irrigation water electrical conductivity from 0.9 to 3.3 dS m^−1^ in the absence of Asc (0 mg L^−1^). The maximum value of 56.34 mm was observed in the control treatment, with an irrigation water electrical conductivity (ECw) of 0.9 dS m^−1^. In contrast, the application of ascorbic acid (Asc) at 600 mg L^−1^ resulted in a 4% reduction compared with the control (0 mg L^−1^). In plants grown under the highest ECw level (3.3 dS m^−1^), the application of Asc at 600 mg L^−1^ increased this parameter by 4.69% compared with plants subjected to the same irrigation salinity level without Asc application (0 mg L^−1^).

**Figure 2:**
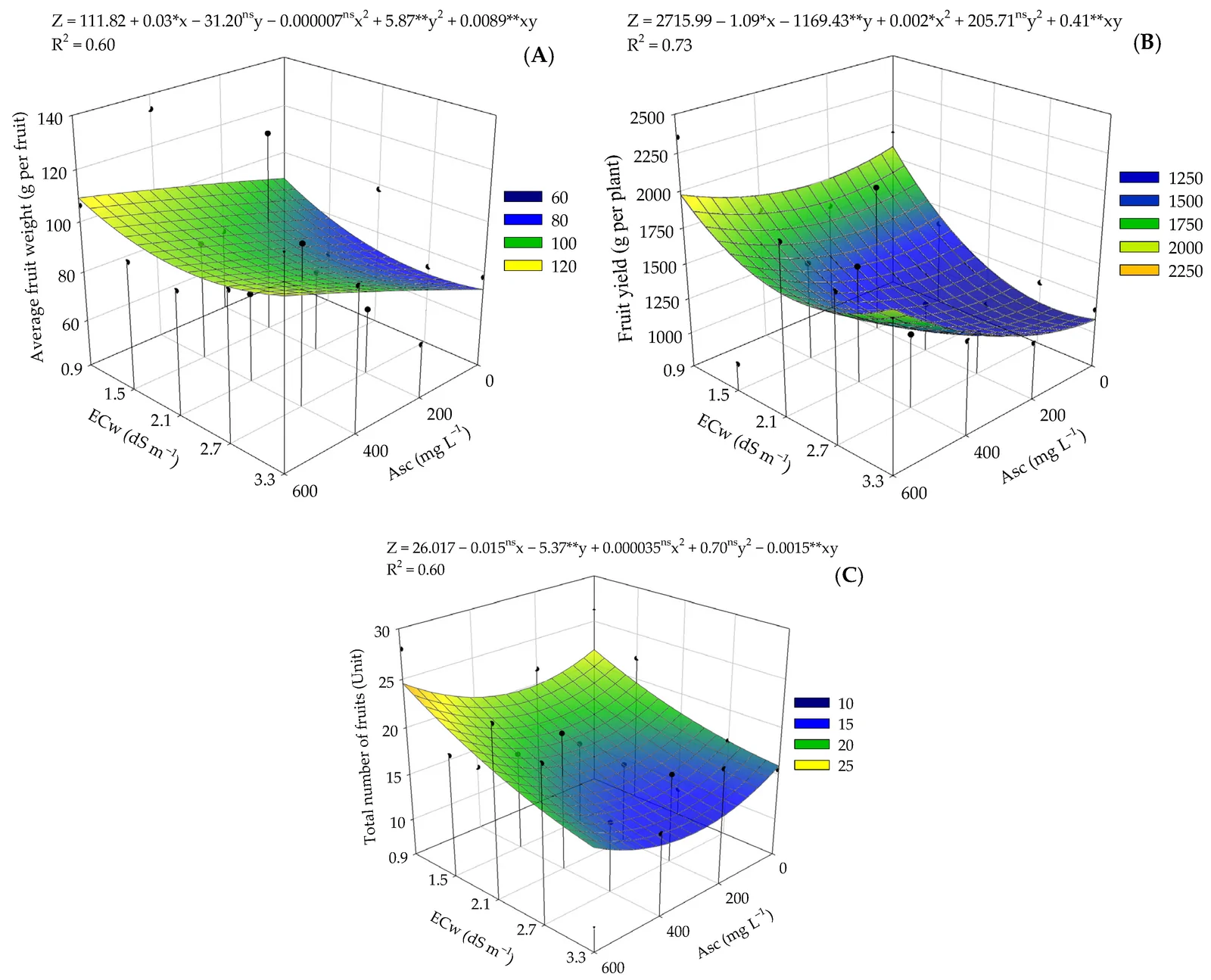
Average fruit weight (AFW) (**A**), fruit yield (FY) (**B**), and total number of fruits (TNF) (**C**) of guava cv. Paluma as affected by the interaction between irrigation water electrical conductivity (ECw) and ascorbic acid concentrations (Asc). X and Y represent ascorbic acid concentration (Asc) and irrigation water electrical conductivity (ECw). ^ns^, not significant (*p* > 0.05); *, ** significant at *p* ≤ 0.05 and *p* ≤ 0.01 by the F-test, respectively.

Increasing irrigation water electrical conductivity (ECw) from 0.9 to 3.3 dS m^−1^ reduced average fruit weight (AFW) (Figure 2A) by 17.75%. In contrast, the application of ascorbic acid (Asc) at 600 mg L^−1^ increased AFW regardless of the irrigation water electrical conductivity level. The highest estimated AFW, 108.80 g fruit^−1^, was obtained in plants irrigated with water at an ECw of 0.9 dS m^−1^ and treated with 600 mg L^−1^ Asc, representing a 22.92% increase compared with the untreated control under the same irrigation conditions. At the same Asc concentration, AFW in plants grown under an ECw of 3.3 dS m^−1^ increased by 45.48% compared with plants irrigated at the same salinity level without Asc application.

Water salinity reduced fruit yield (Figure 2B) by 40.06% as ECw increased from 0.9 to 3.3 dS m^−1^. The application of 600 mg L^−1^ Asc resulted in the highest FY (2117.53 g plant^−1^) in plants irrigated with the lowest water salinity level (0.9 dS m^−1^), representing a 15.70% increase compared with plants without Asc application. Similar results were observed in plants irrigated with the highest ECw, which showed an 80% increase in FY compared with plants subjected to the same irrigation condition without Asc application. In contrast, the model-predicted Asc concentration of 180 mg L^−1^ resulted in a reduction in this variable, with the lowest value (1126.01 g plant^−1^) observed in plants irrigated with water at an ECw of 2.7 dS m^−1^ at this same Asc concentration.

A 26.81% reduction in total number of fruits (TNF) (Figure 2C) was observed as irrigation water electrical conductivity (ECw) increased from the lowest to the highest level in plants that did not receive Asc. With the application of Asc at 600 mg L^−1^, the highest estimated value (24.54 fruits plant^−1^) was recorded in plants irrigated with water at an ECw of 0.9 dS m^−1^, representing a 12.83% increase compared with the control treatment (0 mg L^−1^) under the same irrigation conditions. A similar effect was observed at the highest ECw level, with a 4% increase compared with plants irrigated at the same ECw without Asc application. In contrast, the model-predicted Asc concentration of 230 mg L^−1^ resulted in the lowest TNF (13.18 fruits plant^−1^) under irrigation with water at an ECw of 3.3 dS m^−1^.

### 2.2. Postharvest Quality

The interaction between irrigation water electrical conductivity (ECw) and ascorbic acid (Asc) concentrations significantly affected pulp pH, titratable acidity (TA), vitamin C (Vit. C), and total sugars (TS) of guava cv. Paluma (Table S2). Irrigation water electrical conductivity and ascorbic acid concentrations also significantly influenced the total soluble solids (TSS) content of guava pulp (Table S2).

**Figure 3:**
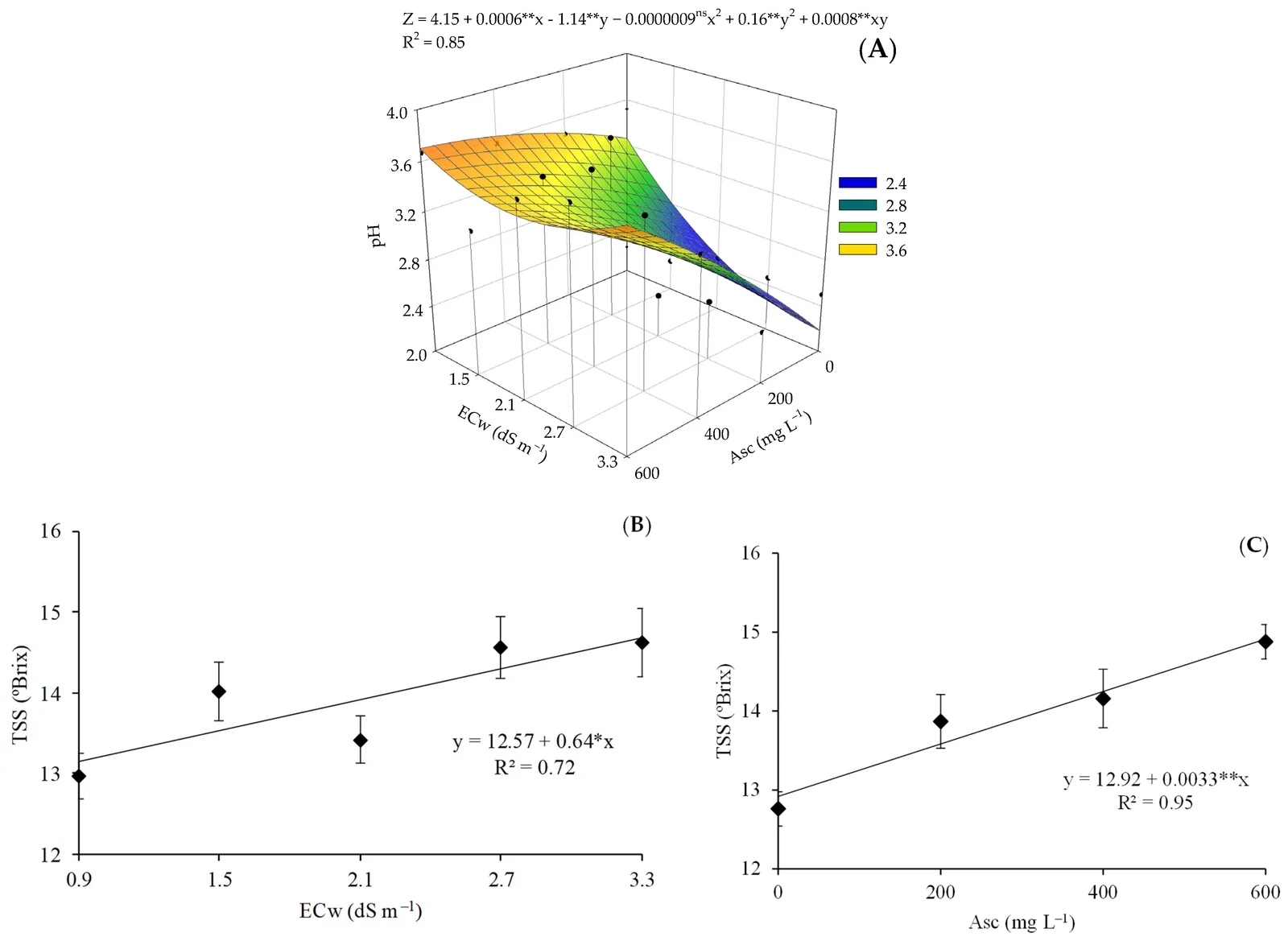
Pulp pH (**A**) of guava cv. Paluma as affected by the interaction between irrigation water electrical conductivity (ECw) and ascorbic acid concentrations (Asc); total soluble solids (TSS) as affected by irrigation water electrical conductivity (ECw) (**B**) and ascorbic acid concentrations (Asc) (**C**). X and Y represent ascorbic acid concentration (Asc) and irrigation water electrical conductivity (ECw). ^ns^, not significant (*p* > 0.05); *, ** significant at *p* ≤ 0.05 and *p* ≤ 0.01 by the F-test, respectively.

Regarding fruit pulp pH (Figure 3A), a 34.52% reduction was observed with the increase in irrigation water electrical conductivity from 0.9 to 3.3 dS m^−1^ in the absence of Asc. Foliar application of ascorbic acid at 600 mg L^−1^ resulted in the highest pH (3.72) in plants irrigated with an ECw of 0.9 dS m^−1^, representing a 14.38% increase compared with plants without Asc application. Conversely, the lowest pH (2.13) was observed in plants irrigated with an ECw of 3.3 dS m^−1^ without foliar Asc application (0 mg L^−1^). However, these effects were mitigated by the application of 600 mg L^−1^ Asc, which resulted in a 76.04% increase compared with plants subjected to the same ECw without Asc application.

Water salinity linearly increased the total soluble solids content (Figure 3B) of guava fruits, with an increase of 5.09% per unit increase in ECw. In relative terms, TSS increased by 11.68% (1.54 °Brix) when comparing plants irrigated with ECw of 3.3 dS m^−1^ and 0.9 dS m^−1^.

Considering total soluble solids (TSS) independently (Figure 3C), the regression model indicated a 15.32% increase (1.98 °Brix) in plants treated with a foliar application of 600 mg L^−1^ Asc compared with plants that did not receive Asc (0 mg L^−1^).

**Figure 4:**
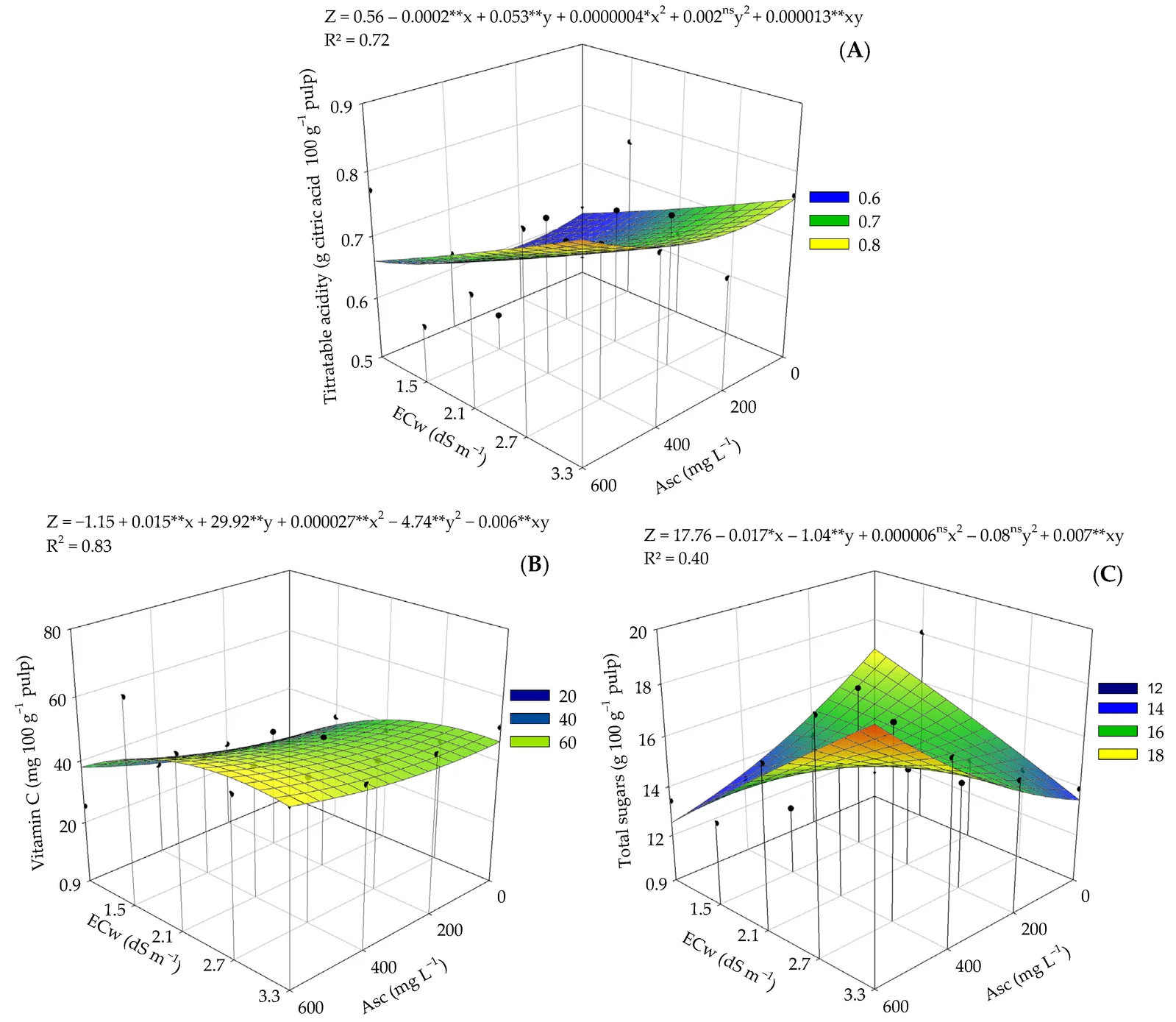
Titratable acidity (TA) (**A**), vitamin C (Vit. C) (**B**), and total sugars (TS) (**C**) in the pulp of guava cv. Paluma as affected by the interaction between irrigation water electrical conductivity (ECw) and ascorbic acid concentrations (Asc). X and Y represent ascorbic acid concentration (Asc) and irrigation water electrical conductivity (ECw). ^ns^, not significant (*p* > 0.05); *, ** significant at *p* ≤ 0.05 and *p* ≤ 0.01 by the F-test, respectively.

Titratable acidity (Figure 4A) increased by 24.18% with increasing ECw levels when comparing plants irrigated with 0.9 dS m^−1^ and those irrigated with 3.3 dS m^−1^. In plants subjected to an ECw of 3.3 dS m^−1^, foliar application of 600 mg L^−1^ Asc resulted in the highest TA value (0.806 g citric acid 100 g^−1^ pulp), representing a 6.57% increase compared with plants irrigated with the same ECw without Asc application (0 mg L^−1^). Conversely, the lowest estimated TA value (0.59 g citric acid 100 g^−1^ pulp) was observed in plants irrigated with water at 0.9 dS m^−1^ and treated with a model-predicted Asc concentration of 200 mg L^−1^.

Regarding vitamin C content (Figure 4B), increasing the irrigation water electrical conductivity from 0.9 to 3.3 dS m^−1^ in the absence of Asc resulted in a 109.53% increase in vitamin C content. Conversely, a beneficial effect of Asc application at 600 mg L^−1^ was observed in plants irrigated with an estimated ECw of 2.8 dS m^−1^, resulting in the highest Vit. C content (54.10 mg 100 g^−1^ pulp), representing a 19% increase compared with plants subjected to the same ECw without Asc application (0 mg L^−1^). It is noteworthy that the lowest Vit. C content (21.93 mg 100 g^−1^ pulp) was observed in plants without Asc application.

Regarding total sugars (Figure 4C) in guava fruits, increasing ECw from 0.9 to 3.3 dS m^−1^ resulted in a 24.54% decrease. Concurrently, foliar application of Asc at 600 mg L^−1^ attenuated the deleterious effects of irrigation with an ECw of 3.3 dS m^−1^, resulting in the highest TS content (19.27 g 100 g^−1^ pulp), with a 43.25% increase compared with plants subjected to the same ECw without Asc application.

A significant interaction between water salinity (ECw) and ascorbic acid (Asc) concentrations was observed for the maturity index (MI), flavonoids (FLA), anthocyanins (ANT), and phenolic compounds (PC) of ‘Paluma’ guava (Table S3).

**Figure 5:**
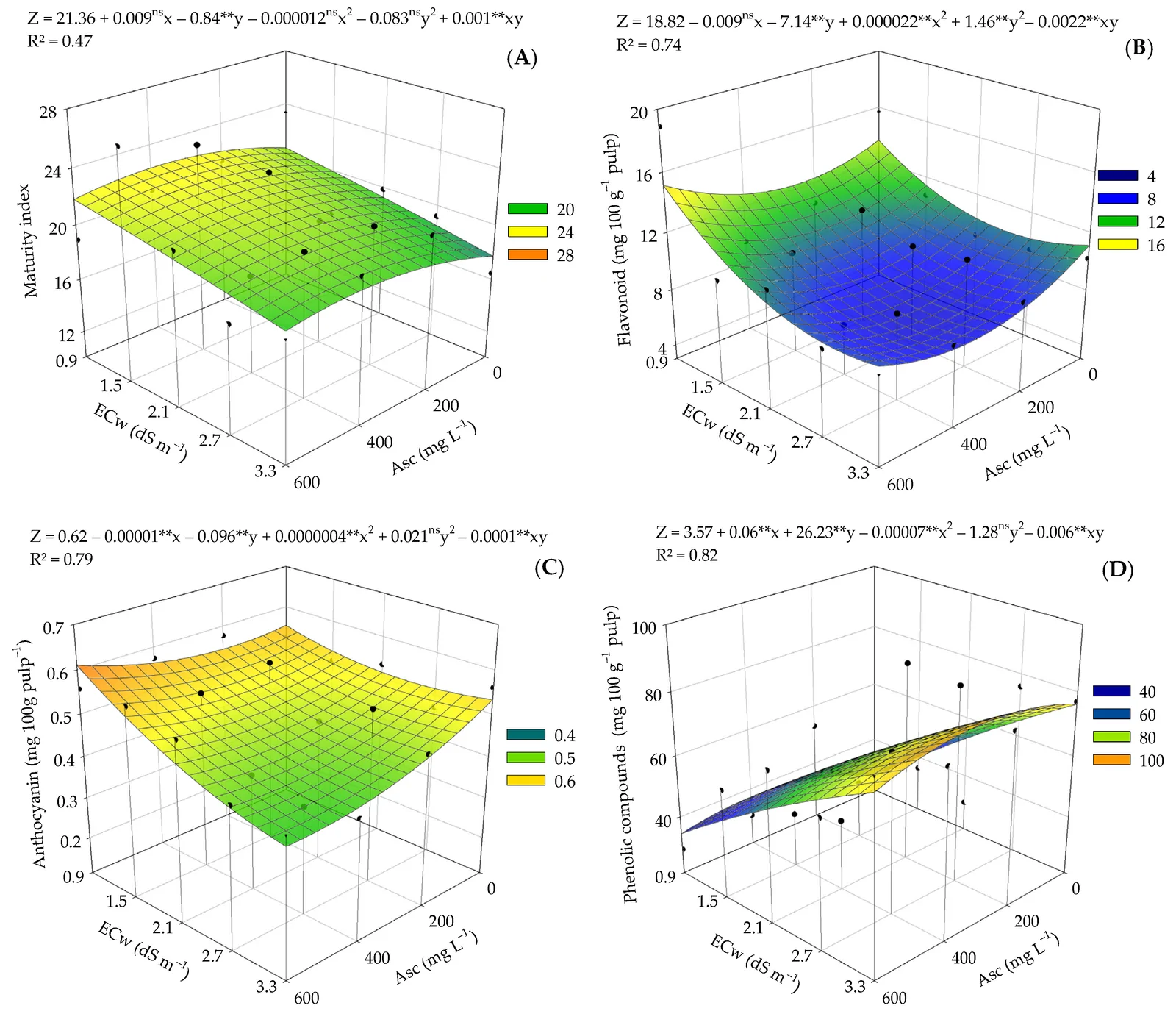
Maturity index (MI) (**A**), flavonoid content (FLA) (**B**), anthocyanin content (ANT) (**C**), and phenolic content (PC) (**D**) in the pulp of guava cv. Paluma, as affected by the interaction between irrigation water electrical conductivity (ECw) and ascorbic acid concentration (Asc). X and Y represent ascorbic acid concentration (Asc) and irrigation water electrical conductivity (ECw). ^ns^, not significant (*p* > 0.05); ** significant at *p* ≤ 0.01 by the F-test.

Increasing ECw reduced the maturity index (Figure 5A) of the fruit pulp by 16.13% when comparing plants irrigated with ECw of 0.9 and 3.3 dS m^−1^. The highest MI value was obtained in plants irrigated with water at an ECw of 0.9 dS m^−1^ and treated with 410 mg L^−1^ Asc, representing a 9.94% increase compared with plants irrigated with the same ECw without Asc application. Conversely, the lowest MI value (17.68) was observed in plants irrigated with water at an ECw of 3.3 dS m^−1^ without Asc application (0 mg L^−1^).

Regarding flavonoid content (Figure 5B), increasing water salinity from 0.9 to 3.3 dS m^−1^ resulted in a 17.82% reduction. Conversely, in plants irrigated with an ECw of 0.9 dS m^−1^, foliar application of 600 mg L^−1^ Asc resulted in the highest FLA content (14.91 mg 100 g^−1^ pulp). However, according to the fitted regression model, an Asc concentration of 380 mg L^−1^ at an ECw of 2.8 dS m^−1^ was associated with the lowest predicted flavonoid content (7.71 mg 100 g^−1^ pulp).

Increasing water salinity from 0.9 to 3.3 dS m^−1^ reduced anthocyanin content (Figure 5C) by 32.58%. In contrast, increasing Asc concentration from 0 (without Asc application) to 130 mg L^−1^, combined with irrigation at an ECw of 3.3 dS m^−1^, resulted in the lowest predicted anthocyanin content (0.33 mg 100 g^−1^ pulp), representing an 11.17% reduction compared with plants grown under the same ECw without Asc application. However, the highest anthocyanin content (ANT; 0.64 mg 100 g^−1^ pulp) was observed in plants irrigated with water at an ECw of 0.9 dS m^−1^ and treated with 600 mg L^−1^ Asc.

Phenolic compound content (Figure 5D) increased by 191.47% as ECw increased from 0.9 to 3.3 dS m^−1^. The highest value (82 mg 100 g^−1^ pulp) observed was estimated by the regression model in plants irrigated with an ECw of 3.3 dS m^−1^ and treated with 290 mg L^−1^ Asc, representing a 7.57% increase compared with plants subjected to the same ECw without Asc application. The lowest PC was observed in control plants irrigated with an ECw of 0.9 dS m^−1^ without Asc application (0 mg L^−1^), representing a 27.56% reduction compared with plants treated with the same ECw and predicted by the model at 290 mg L^−1^ Asc.

### 2.3. Pearson Correlation

**Figure 6:**
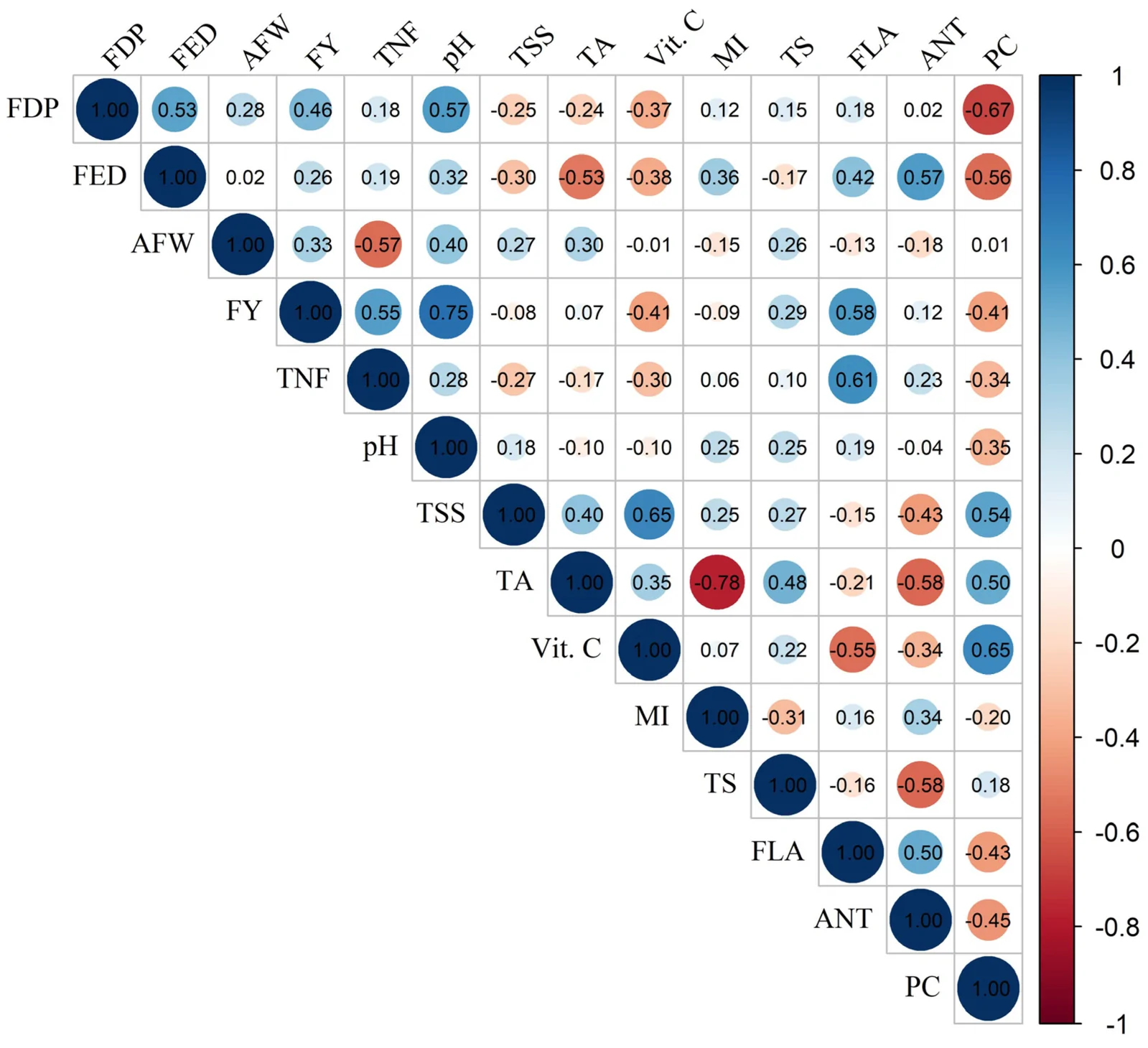
Pearson correlation matrix of yield components and postharvest quality attributes of guava cv. Paluma grown under different irrigation water electrical conductivity levels (ECw) and ascorbic acid concentrations (Asc).

The relationships among yield components and postharvest quality attributes of guava cv. Paluma under different irrigation water electrical conductivity levels and ascorbic acid concentrations are presented in the Pearson correlation matrix (Figure 6). Fruit polar diameter showed a positive correlation with equatorial diameter (r = 0.53) and pH (r = 0.57). Conversely, it showed a moderate negative correlation with phenolic compounds (r = −0.67). A similar effect was observed between equatorial diameter and phenolic compounds (r = −0.56).

Yield showed a strong positive correlation with pH (r = 0.75) and a moderate positive correlation with flavonoid content (r = 0.58). The postharvest quality variables showed distinct correlation patterns. Negative correlations were observed between titratable acidity (TA) and anthocyanins (ANT) (r = −0.58), and between vitamin C (Vit. C) and flavonoids (FLA) (r = −0.55). Positive correlations (r > 0.50) were observed among phenolic compounds (PC), total soluble solids (TSS), titratable acidity (TA), and vitamin C (Vit. C).

### 2.4. Network Analysis

**Figure 7:**
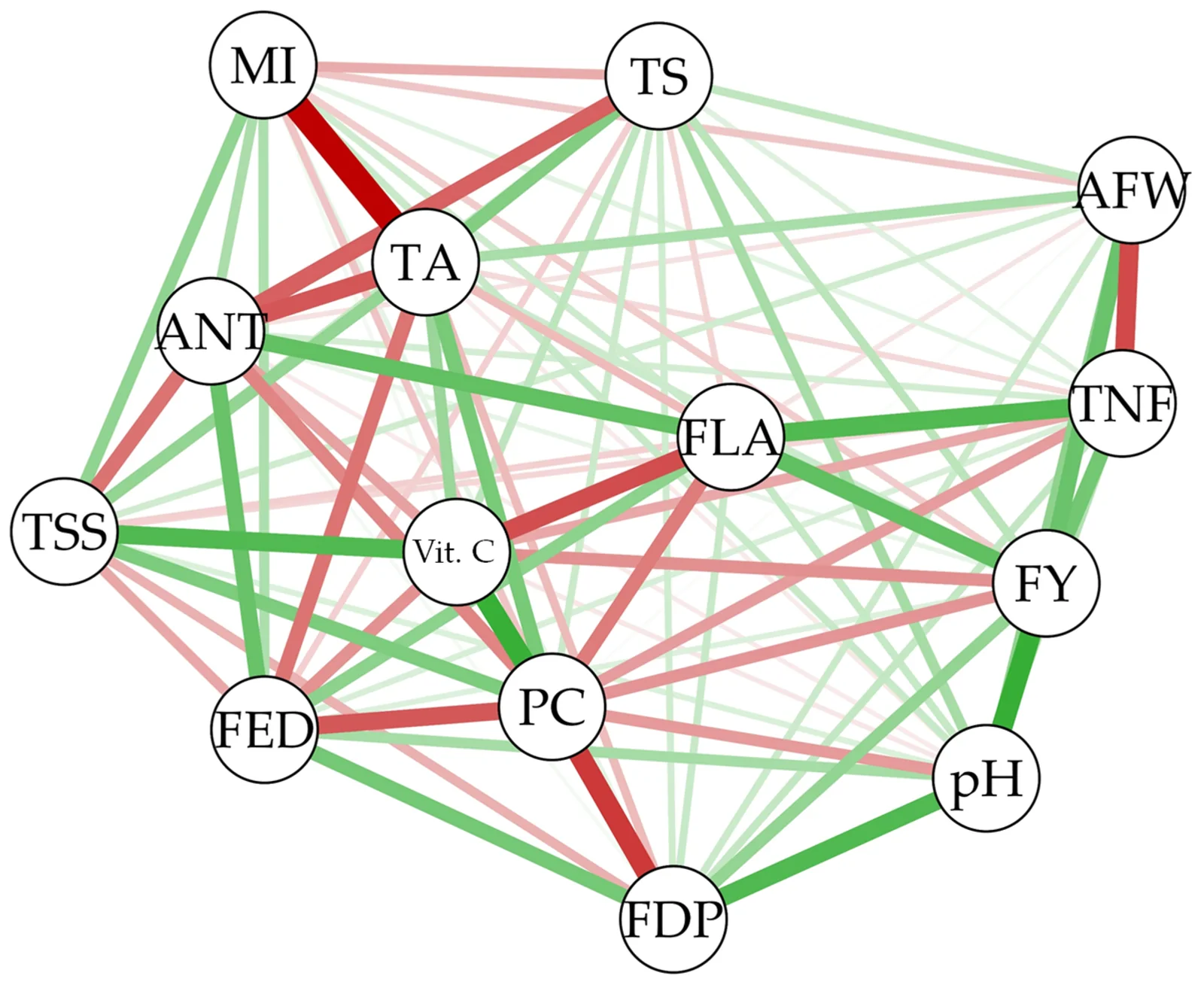
Network analysis of the variables evaluated under the interaction between ascorbic acid concentrations (Asc) and irrigation water electrical conductivity (ECw). Red edges indicate negative associations, whereas green edges indicate positive associations. Node size indicates connectivity within the network, whereas edge thickness represents the magnitude of the correlations. Green edges indicate positive correlations, while red edges indicate negative correlations; thicker edges represent stronger associations. The connections are undirected and therefore do not represent causal relationships.

The patterns observed in the Pearson correlation matrix (Figure 6) were corroborated by the network analysis (Figure 7). Variables positioned closer to each other exhibited stronger correlations, whereas greater distances between nodes indicated weaker associations. In addition, edge thickness represents the strength of the correlations, while green and red edges denote positive and negative associations, respectively, among the variables evaluated.

Overall, yield components, fruit physical characteristics, and chemical quality attributes exhibited distinct patterns of association in the network analysis. The maturity index showed a strong negative association with titratable acidity and total sugars, suggesting that fruit maturation was accompanied by a reduction in acidity. However, this process was not associated with an increase in total sugar content, indicating that fruit ripening did not result in greater accumulation of these compounds.

Yield components were positively associated with the physical characteristics of the fruits, indicating that these traits varied in an integrated manner and reflecting greater uniformity in fruit development. In contrast, average fruit weight (AFW) showed a negative association with the total number of fruits (TNF), suggesting a potential trade-off between these yield components, whereby an increase in fruit number was associated with a reduction in individual fruit weight.

In contrast, yield components and fruit physical characteristics were predominantly negatively associated with postharvest quality attributes, except for pH, flavonoid content, total sugar content, and anthocyanin content. This pattern suggests a potential trade-off between yield components and certain chemical attributes, indicating that larger fruits or those produced by more productive plants tended to exhibit lower titratable acidity without compromising the accumulation of bioactive compounds or sugars.

### 2.5. Multi-Trait Genotype-Ideotype Distance Index (MGIDI)

Principal component analysis (PCA) (Table 1), performed based on the multi-trait genotype–ideotype distance index (MGIDI), identified five principal components (PC1–PC5) with eigenvalues ≥ 1, which collectively explained 85.43% of the total experimental variance. Considering the correspondence between principal components and factors (PC = FA), five factors (FA1–FA5) were obtained. A significant interaction between irrigation water electrical conductivity (ECw) and ascorbic acid (Asc) concentrations was detected for the set of traits evaluated, as indicated by the likelihood ratio test (*p* ≤ 0.01).

**Figure 8:**
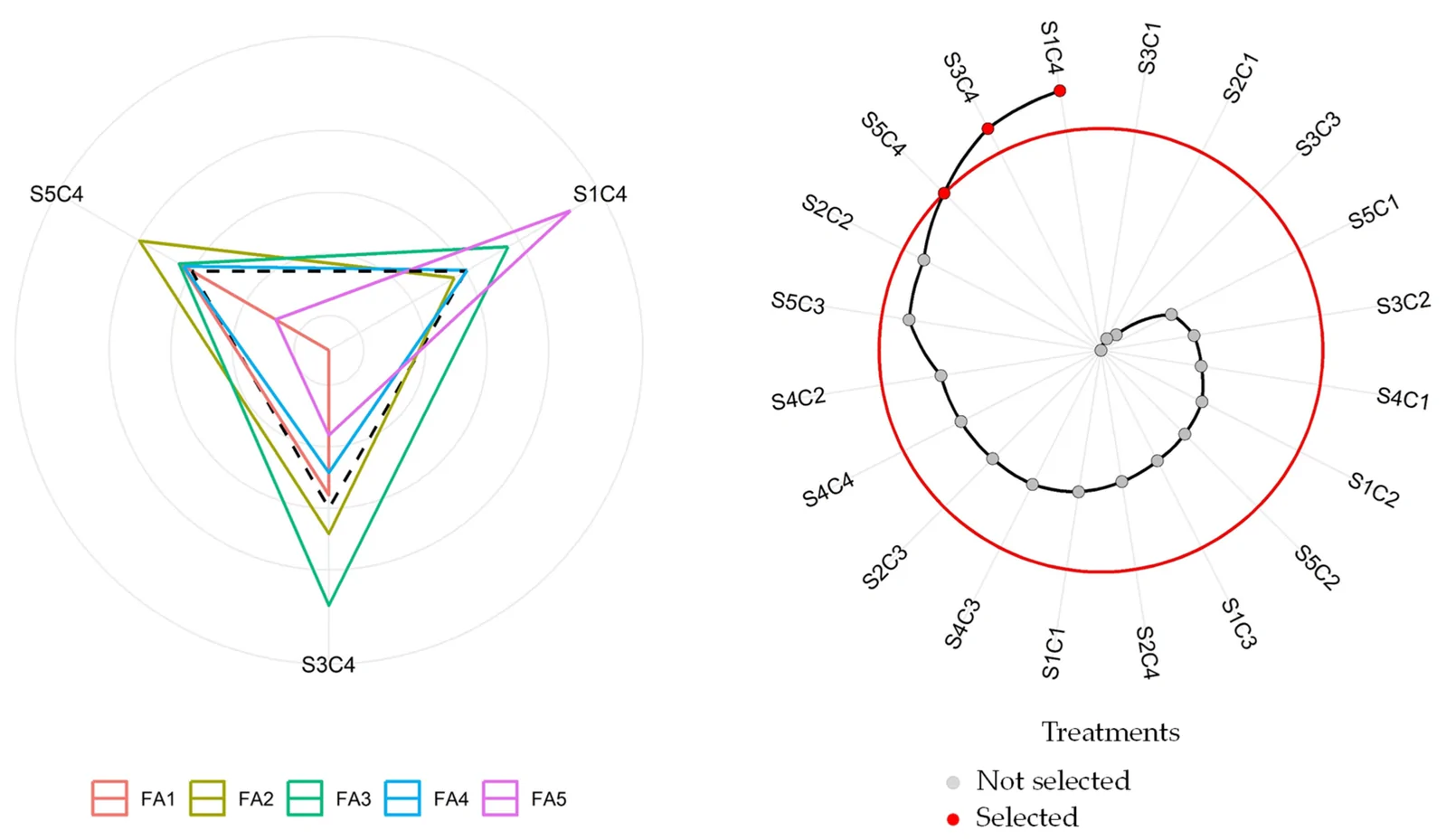
Ranking of treatments based on the Multi-Trait Genotype–Ideotype Distance Index (MGIDI) under the interaction between irrigation water electrical conductivity (ECw) and ascorbic acid (Asc) concentrations (ECw × Asc).

The treatments S1C4, S3C4, and S5C4 were the closest to the ideotype (Figure 8), indicating superior overall performance and stability. Under irrigation with an ECw of 0.9 dS m^−1^ and foliar application of 600 mg L^−1^ Asc (S1C4), the greatest contributions were associated with FA1 (45.60%), followed by FA2 (22.20%), FA4 (19.70%), FA3 (12.10%), and FA5 (0.40%). In plants irrigated with an ECw of 2.1 dS m^−1^ and treated with 600 mg L^−1^ Asc (S3C4), the highest contributions were observed for FA5 (31.90%), FA4 (25.80%), FA1 (22.20%), FA2 (15.90%), and FA3 (4.26%). Likewise, plants irrigated with an ECw of 3.3 dS m^−1^ and sprayed with 600 mg L^−1^ Asc (S5C4) showed the greatest association with FA5 (35.70%), followed by FA4 (18.40%), FA1 (18.20%), FA3 (17.60%), and FA2 (10.20%).

The analysis of the traits grouped within the MGIDI factors (Table 2) revealed that the interaction between irrigation water electrical conductivity (ECw) and ascorbic acid (Asc) concentrations promoted integrated responses in yield components and the physical and chemical attributes of the fruits. These factors made it possible to identify the traits that contributed most to the proximity of each treatment to the ideotype.

In this context, parameters such as repeatability indicate the consistency of responses observed across repeated evaluations, allowing an estimate of how much of the observed performance is likely to persist, whereas selection gains represent the expected change in the mean performance of traits following the selection of the most favorable treatments.

FA1 comprised traits related to the chemical quality of the fruits, including total soluble solids (TSS), vitamin C (Vit. C), and phenolic content (PC). The relative responses predicted by the MGIDI were 5.26%, 5.18%, and 3.99%, respectively, and were associated with high repeatability estimates (h^2^ = 0.82–0.97). This factor contributed most under the treatment involving irrigation with water at an ECw of 0.9 dS m^−1^ and foliar application of 600 mg L^−1^ Asc. FA2 grouped fruit polar diameter and pulp pH, representing traits associated with the physical and chemical quality of the fruits. The predicted relative responses were 3.55% for fruit polar diameter and 14.70% for pH, both associated with high repeatability estimates (h^2^ = 0.93–0.97).

FA3 was the most representative factor, comprising the largest number of evaluated traits. Within this group, the highest predicted relative responses were observed for titratable acidity (TA), total sugars (TS), and anthocyanin content (ANT), with values of 9.24%, 3.28%, and 1.60%, respectively, and a mean repeatability estimate of h^2^ = 0.86. In addition, fruit equatorial diameter (FED) and maturity index (MI) showed predicted relative responses of −0.74% and −3.25%, respectively, indicating contrasting responses of these traits relative to the ideotype and suggesting a possible trade-off between fruit growth and quality-related attributes.

FA4 comprised the yield components, represented by average fruit weight (AFW) and total number of fruits (TNF). The predicted relative responses of 29.90% and 11.80%, respectively, together with a mean repeatability estimate of h^2^ = 0.84 across the evaluated treatments, indicate that this factor was strongly associated with traits contributing to the productive performance of the treatments. FA5 grouped fruit yield (FY) and flavonoid content (FC), integrating productive performance with the functional quality of the fruits. The predicted relative responses of 28.60% for fruit yield and 23.80% for flavonoid content, together with repeatability estimates of 0.77 and 0.98, respectively, indicate that this factor was associated with a set of traits that contributed simultaneously to productivity and the accumulation of bioactive compounds.

Factors FA4 and FA5 made their greatest contributions under treatment S5C4. This pattern may be associated with the beneficial effects of Asc, since, even under the highest salinity level evaluated (3.3 dS m^−1^), foliar application of 600 mg L^−1^ Asc kept this treatment close to the ideotype. However, this proximity resulted from the combined responses of the evaluated traits to the saline environment and Asc application, rather than providing evidence of genetic selection or genetic gain, because the analysis was designed to evaluate treatments. Furthermore, the set of traits associated with favorable performance under S5C4 differed from that observed at lower salinity levels, indicating that the combination of salinity and Asc application altered the multivariate response pattern of the plants.

In the MGIDI analysis, the Direction column indicates the desired direction of change for each trait (increase or decrease), whereas the Goal column defines the target value assigned to each trait for ideotype construction. In the present study, yield and postharvest quality traits were assigned an increasing direction, as improvements in these attributes are directly associated with greater adaptation to saline conditions. A target value of 100 was assigned to these desirable traits, indicating their maximum contribution to the multivariate selection process. In contrast, fruit equatorial diameter (FED) and maturity index (MI) were assigned a target value of 0 because these traits were not considered priority criteria for defining the ideal ideotype, thereby preventing them from exerting excessive influence on the MGIDI.

Based on these selection criteria, favorable predicted gains were obtained for total soluble solids (TSS), vitamin C (Vit. C), phenolic content (PC), fruit polar diameter (FPD), pulp pH, titratable acidity (TA), total sugars (TS), anthocyanin content (ANT), average fruit weight (AFW), total number of fruits (TNF), fruit yield (FY), and flavonoid content (FLA). These results demonstrate that the selection strategy was effective in simultaneously improving fruit physicochemical quality and productive performance. In this context, the selection gain (SG) quantifies the direction and magnitude of the response to selection relative to the original population mean (X_0_). Positive values indicate gains in desirable traits, whereas negative values represent intentional reductions in traits for which increases were not prioritized in the definition of the ideotype.

Overall, the predicted gains for yield components and fruit physicochemical quality traits indicate that the treatments closest to the ideotype exhibited a balanced combination of desirable responses. These findings reinforce the effectiveness of the MGIDI in integrating multiple traits to identify the most promising treatment combinations under different irrigation water electrical conductivity levels (ECw) and ascorbic acid (Asc) concentrations.

## 3. Discussion

In the present study, reductions were observed in fruit polar and equatorial diameters, average fruit weight, yield, total fruit number, maturity index, and flavonoid and anthocyanin contents in guava fruits when plants were irrigated with brackish water. Irrigation water electrical conductivity reduced both fruit polar diameter (FPD) and equatorial diameter (FED). These results may be associated with the osmotic effect of excess dissolved salts in the soil solution, which increases soil osmotic pressure and makes it more difficult for plants to absorb water and nutrients, even when water is frequently available [21]. In addition, excess salts may promote ionic competition between Na^+^ and Ca^2+^ for root absorption pathways, inhibiting the uptake of this macronutrient [22]. This may directly affect the formation of calcium pectates, which are structural components of the cell wall and consequently influence fruit growth and expansion [23]. These results corroborate those reported by Lacerda et al. [15] in a study with ‘Paluma’ guava irrigated with water at ECw of 0.6 and 3.2 dS m^−1^, in which an ECw of 3.2 dS m^−1^ negatively affected fruit polar and equatorial diameters.

However, the application of ascorbic acid at higher concentrations was associated with improvements in guava yield components under saline irrigation, as reflected by increases in fruit polar and equatorial diameters, average fruit weight, and fruit yield. These results may be associated with the action of ascorbic acid in modulating the biosynthesis of enzymes involved in the production of essential metabolites, such as hydroxyproline, anthocyanins, ethylene, and gibberellins, which, in turn, may be involved in processes associated with fruit growth and consequently result in heavier fruits [24,25]. These findings are consistent with the moderate positive correlation observed between fruit equatorial diameter and yield.

During September, October, and November, which coincided with the flowering stage, the highest temperatures of the year were recorded, reaching 35.6, 36.4, and 36.5 °C, respectively, along with the lowest precipitation levels (0, 1.4, and 0 mm, respectively). Under these conditions, increases in temperature and reductions in relative air humidity may increase flower abortion rates and reduce pollen viability [26]. In addition, considering these conditions, the combined effects of salinity and heat stress may have contributed to the reduction in the number of guava fruits.

Similar results were reported by Ullah et al. [27] in a study investigating the effects of heat stress on wheat physiology, in which the flowering stage was directly affected by increasing temperature. The authors highlighted that these effects may be associated with limited transport of photoassimilates to developing reproductive organs, resulting in smaller grains, impaired pollination, and consequently reduced overall yield.

Regarding postharvest quality, a reduction in pH was observed with increasing ECw. A similar reduction in guava pulp pH with increasing salinity levels was reported by Lima et al. [28] in acerola plants grown under different salinity levels (0.6, 1.4, 2.2, 3.0, and 3.8 dS m^−1^), in which a 9.86% reduction was observed when comparing the lowest and highest salinity levels. Under optimal growing conditions, K^+^ accumulates in the vacuoles, contributing to the neutralization of negative charges associated with organic acids [29].

However, under salt stress conditions, increased Na^+^ concentrations may alter cellular ionic balance and promote K^+^ redistribution among plant tissues. K^+^ plays an important role in maintaining ionic homeostasis and cellular acid–base balance, thereby contributing to pH stability in plant tissues [30]. According to Normative Instruction No. 49 of 26 September 2018, the pH values obtained in this study under moderate salinity levels comply with the standard established by the Brazilian Ministry of Agriculture (3.5) for guava pulp [31].

The increase in soluble solids in response to higher irrigation water electrical conductivity may be associated with a reduction in fruit water content, resulting in a higher concentration of these compounds. Although these mechanisms were not evaluated in the present study, previous studies [32,33] have reported that salt stress may promote the accumulation of sugars and amino acids, which act as compatible osmolytes and contribute to cellular osmotic adjustment. This process may contribute to the increased concentration of soluble solids in fruits.

On the other hand, the increases in soluble solids observed with Asc application may be related to the protection of the plant biochemical machinery. Ascorbic acid contributes to maintaining cell membrane integrity, thereby favoring the maintenance and accumulation of organic solutes involved in osmotic adjustment [19,34]. Despite the variations in soluble solids content resulting from saline irrigation and Asc application, all values remained above the minimum threshold established by Normative Instruction No. 49 of 26 September 2018 for guava pulp, which specifies a minimum soluble solids content of 7 °Brix [31]. This variable reflects both plant performance and the nutritional quality of fruits under the adopted management conditions. This response may also be related to the positive correlations observed in the Pearson correlation matrix between vitamin C and TSS and between vitamin C and phenolic compounds, suggesting that exogenous Asc application not only helped preserve cell integrity but may also have improved the accumulation of osmolytes derived from sugars and secondary antioxidants [35].

The titratable acidity (TA) results are consistent with those reported by Ferreira et al. [12] in a study conducted with guava plants to evaluate the effects of irrigation strategies using saline water and ascorbic acid application (0, 200, 400, and 600 mg L^−1^), in which the authors observed increases in TA with increasing ascorbic acid concentrations. Foliar-applied ascorbic acid may directly contribute to the TA values obtained, as it is an acid and participates in the neutralization reaction during titration. In this regard, the increase in TA observed following Asc application may reflect both the contribution of the applied compound. Although these responses may be associated with treatment effects on acid metabolism and the physiological balance of the plants [36], these mechanisms were not directly evaluated in the present study. According to Normative Instruction No. 49 of 26 September 2018, issued by the Brazilian Ministry of Agriculture, the results obtained in this study fall within the range considered suitable for guava pulp, for which a minimum TA of 0.4 g 100 g^−1^ is specified [31].

An inverse relationship was observed between TA and the maturity index (MI) in the Pearson correlation matrix, whereby reductions in acidity may be directly associated with increases in the maturity index, reflecting the progression of fruit ripening [37]. However, TA increased with increasing Asc concentrations, a pattern also supported by the principal component analysis. In the latter, TA was associated with Cluster 5, which comprised treatments combining the highest ECw levels and Asc concentrations, introducing the hypothesis that these factors may act in the retention or synthesis of organic acids in the fruit.

Asc plays an important role in cellular redox homeostasis [18] and may contribute to the nutritional quality of guava fruits. According to Normative Instruction No. 49 issued by the Brazilian Ministry of Agriculture [31], the values obtained in this study, even under high salinity conditions, exceeded the minimum standard of 24 mg 100 g^−1^ established for guava pulp. However, it is important to note that vitamin C content was determined by DCPIP titration following extraction with oxalic acid. Although widely used, this method is based on reducing capacity and may be affected by interference from other reducing substances present in the plant matrix. Lacerda et al. [15], for example, reported vitamin C contents of up to 143.94 mg 100 g^−1^ in ‘Paluma’ guava fruits grown under low salinity conditions (0.6 dS m^−1^).

Nevertheless, direct comparisons between these values and those obtained in the present study should be made with caution, since differences in cultivar or rootstock, environmental conditions, fruit maturity stage, and analytical procedures may contribute to the observed variation. In addition, salt stress may alter carbon metabolism and resource allocation toward osmotic adjustment and ionic homeostasis, potentially influencing the biosynthesis and accumulation of ascorbic acid in fruits [16]. Since these mechanisms were not directly evaluated in the present study, this explanation should be considered a physiological hypothesis.

The increase in sugar content observed following Asc application in guava plants under salt stress may be associated with the role of Asc in the cytosol as a coenzyme involved in organic molecule metabolism, potentially regulating ATP and NADPH production during the Calvin cycle even under stress conditions, thereby improving carbon assimilation and, consequently, sugar formation [38]. The total sugar (TS) values obtained in the present experiment are consistent with those reported by Ferreira et al. [12] for guava plants treated with Asc, who recorded a maximum value of 14.95 mg 100 g^−1^ pulp in plants irrigated with water at an ECw of 3.3 dS m^−1^ during the vegetative/fruiting stage and treated with 600 mg L^−1^ Asc. Similarly, the present study showed a beneficial effect on TS in plants irrigated with an ECw of 3.3 dS m^−1^ and treated with 600 mg L^−1^ Asc.

In postharvest management, the maturity index is determined by continuously evaluating the physical and chemical changes that characterize the fruit ripening process. The increases observed in the maturity index (MI) may be associated with the action of Asc in protecting lipids and proteins in cell walls against oxidative damage caused by salt stress [39]. Thus, organic sugars can be stored in the vacuoles. In addition, foliar application of Asc may act as a precursor in biosynthetic pathways, serving as a cofactor for 1-aminocyclopropane−1-carboxylic acid oxidase (ACC oxidase), which catalyzes the final step in the synthesis of the plant hormone ethylene during fruit ripening [40,41], particularly in plants subjected to salt stress.

In the present study, Asc application reduced flavonoid content as irrigation water electrical conductivity increased. In contrast, Gaafar et al. [42], in a study of common bean (*Phaseolus vulgaris* L.) under water deficit, reported that Asc application at 400 mg L^−1^ increased the accumulation of flavonoids and phenolic compounds [43,44]. These differences suggest that the effects of Asc application on plant metabolism may vary depending on experimental conditions, stress intensity, and the plant species evaluated.

Anthocyanins are responsible for the red and purple pigmentation of plant tissues. They are synthesized in the cytosol, subsequently converted into other secondary derivatives, and transported to the vacuole [45]. Their synthesis is primarily modulated in response to abiotic stresses, such as salinity [46]. However, the results of the present study indicate that increasing irrigation water electrical conductivity reduced flavonoid accumulation in the fruit pulp under the experimental conditions. Although flavonoids contribute to plant antioxidant defense, salt stress may impair their biosynthesis [47], which could partly explain why the highest anthocyanin content (ANT) was observed in the control treatment.

In guava fruits, phenolic compounds, which play an important role in the antioxidant system, increased with increasing irrigation water electrical conductivity (ECw). According to Mansour and Hassan [47], this response may be associated with the ability of plants to acclimate to stress by increasing the biosynthesis of secondary metabolites and regulating osmotic balance. Furthermore, because glucose participates in metabolic pathways involved in phenolic compound synthesis, the increase in soluble solids may indicate greater availability of substrates potentially used in this biosynthetic process [48]. This hypothesis is also supported by the Pearson correlation matrix, which showed positive correlations between total soluble solids (TSS) and phenolic compounds (PC; r = 0.54) and between vitamin C and PC (r = 0.65), suggesting increased phenolic compound accumulation under stress conditions.

In summary, the present study demonstrated that foliar application of ascorbic acid (Asc) as a potential salinity stress-mitigating agent may provide an alternative for enabling the use of brackish water in grafted guava cultivation using the ‘Paluma’ cultivar grafted onto the BRS Guaraçá hybrid rootstock. Under these conditions, quality attributes such as pH, soluble solids, vitamin C, and titratable acidity remained within the standards considered acceptable for domestic commercialization. However, further studies are recommended to evaluate Asc application at concentrations above 600 mg L^−1^, assess the antioxidant system, extend the evaluation over more than one growing cycle, and conduct field trials to clarify its role in the physiology of plants under salt stress.

According to the MGIDI, treatments S1C4, S3C4, and S5C4 showed the greatest proximity to the ideotype, indicating superior performance compared with the other combinations of irrigation water electrical conductivity levels and ascorbic acid concentrations. This result demonstrates that the simultaneous integration of multiple traits enables the identification of treatments capable of optimizing fruit yield and quality [49]. In this context, the MGIDI constitutes a robust tool for multi-trait selection, as it integrates yield and fruit quality attributes into a single metric. This approach reduces the subjectivity inherent in selection based on individual traits, enabling a more comprehensive and balanced assessment of treatment performance and increasing the reliability of identifying the most promising combinations [50].

## 4. Materials and Methods

### 4.1. Experiment Location

**Figure 9:**
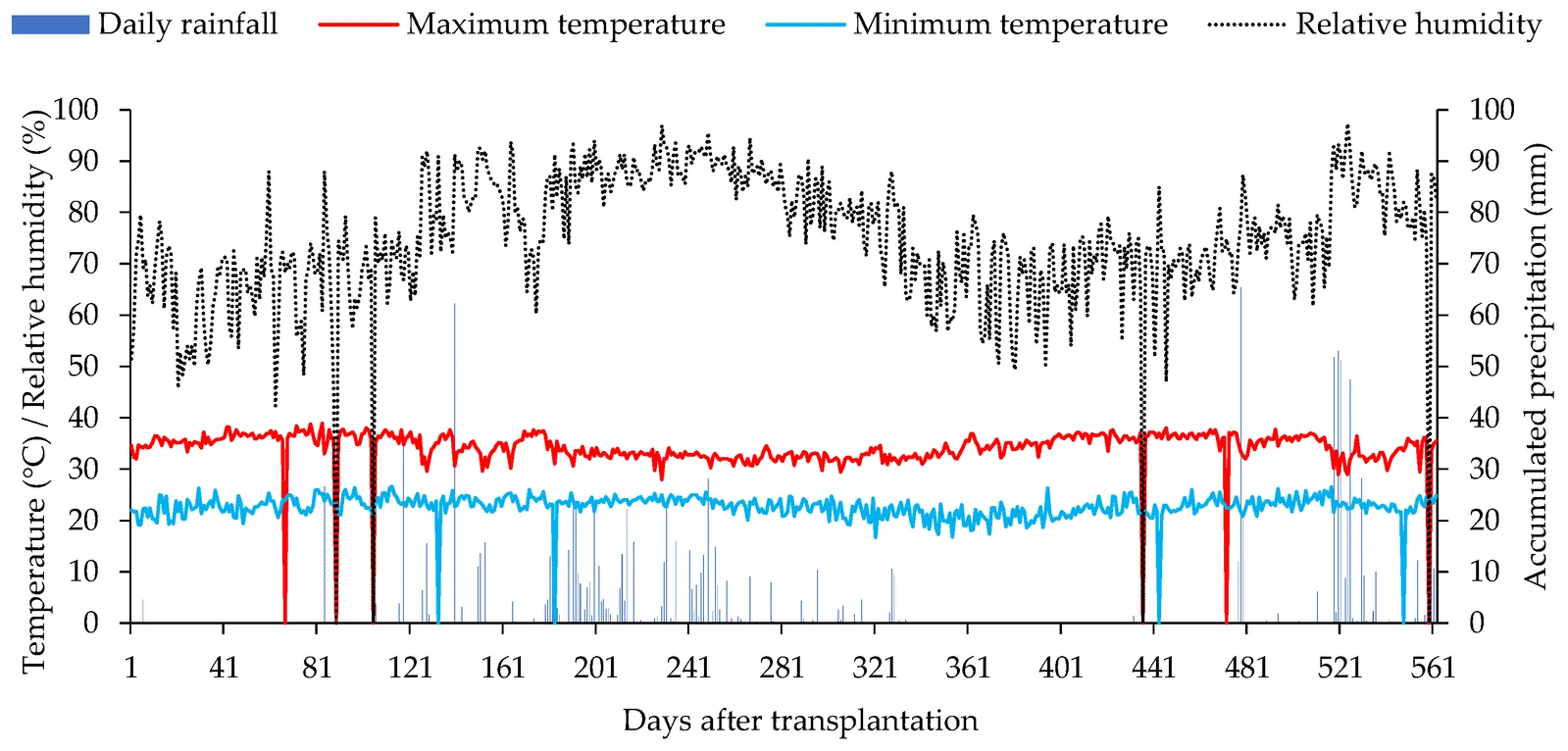
Daily records of maximum and minimum air temperature, relative humidity, and cumulative precipitation collected from 15 August 2023 to 26 February 2025.

The experiment was conducted under field conditions using drainage lysimeters over two consecutive production cycles. Total yield was calculated as the sum of production from August 2023 to February 2025. The study was carried out at the ‘Rolando Enrique Rivas Castellón’ Experimental Farm, belonging to the Center for Food Science and Technology (CCTA) of the Federal University of Campina Grande (UFCG), located in São Domingos, Paraíba, Brazil (6°48′50″ S, 37°56′31″ W; 190 m above sea level). Guava plants were cultivated in 60 L plastic pots adapted as drainage lysimeters. Meteorological data, including maximum and minimum air temperature, relative humidity, and cumulative precipitation, were obtained from the weather station located in the São Gonçalo district, Sousa, Paraíba, Brazil, and are presented in Figure 9.

### 4.2. Experimental Design and Treatments

The experiment was conducted using a randomized complete block design in a split-plot arrangement, with five irrigation water electrical conductivity levels (ECw: 0.9, 1.5, 2.1, 2.7, and 3.3 dS m^−1^) assigned to the main plots and four ascorbic acid (Asc) concentrations (0, 200, 400, and 600 mg L^−1^) assigned to the subplots, with three replicates and one plant per experimental plot. The ECw levels and Asc concentrations were established based on the study conducted by Torres et al. [19]. The plants were randomly distributed within each block, ensuring experimental randomization. All plants within each experimental unit were arranged at a spacing of 2.5 m between plants and 3.0 m between rows. The plants were grown in lysimeters measuring 55 cm in height, 35 cm in internal diameter, and 40 cm in base diameter, with a capacity of 75 kg of soil.

### 4.3. Cultivar Used and Acquisition of Seedlings

The seedlings were propagated by grafting. The scion consisted of the guava cultivar *Paluma*, which produces pyriform fruits with an average weight exceeding 200 g, intense red flesh, and excellent sensory quality [51]. The rootstock used was the hybrid cultivar *BRS Guaraçá*, which combines characteristics of guava (*Psidium guajava* L.) and araçá (*Psidium guineense* Sw.), and is characterized by resistance to root-knot nematodes, as well as high graft compatibility and vigorous plant growth [52]. The grafted seedlings were transplanted 100 days after grafting, when they had reached an average height of approximately 30 cm.

### 4.4. Experimental Setup

The drainage lysimeters used in the experiment were perforated at the base to allow drainage and fitted with a transparent 16 mm diameter drain tube. The inner end of the drain was surrounded by a layer of sand to prevent clogging by soil particles. A plastic container was placed beneath each drain to collect the drainage water, allowing the determination of plant water consumption.

Initially, each lysimeter was filled with a 0.5 kg layer of gravel (No. 0) at the bottom, near the drainage outlets, to facilitate water drainage. The lysimeters were then manually filled with 70 kg of eutrophic Fluvisol with a sandy loam texture, collected from the 0–40 cm soil layer at the CCTA/UFCG Experimental Farm. Prior to the establishment of the experiment, the soil was characterized for its chemical and physical hydraulic properties (Table 3) according to the methodology described by Teixeira et al. [53].

### 4.5. Fertilizer Management

Nitrogen, phosphorus, and potassium fertilization was performed according to the recommendations of Cavalcanti [54], starting at 40 days after sowing (DAS), with applications of 100 g of N, 80 g of P_2_O_5_, and 50 g of K_2_O per plant. Fertilizer rates were established based on the crop’s nutritional requirements and soil nutrient availability, with applications split throughout the growing season. Nitrogen fertilization was divided into weekly applications, whereas potassium fertilization was applied in split doses. Phosphorus fertilization was applied at planting. Urea (45% N), monoammonium phosphate (50% P_2_O_5_ and 11% N), and potassium sulfate (50% K_2_O) were used as the respective sources of nitrogen, phosphorus, and potassium. To meet the micronutrient requirements, foliar applications of Dripsol Micro^®^ (Vitas Brasil, Candeiras, Brazilian) were performed every 15 days, beginning at 20 days after transplanting (DAT), by spraying both the adaxial and abaxial leaf surfaces with a solution containing 2 g L^−1^ of the commercial product (1.2% Mg, 0.85% B, 3.4% Fe, 4.2% Zn, 3.2% Mn, 0.5% Cu, and 0.06% Mo).

### 4.6. Water Preparation and Irrigation Management

The irrigation water used for the lowest salinity treatment (ECw = 0.90 dS m^−1^) was obtained from an artesian well (Well 1). The remaining ECw levels were prepared by either diluting the water or adding NaCl to water with an electrical conductivity of 2.66 dS m^−1^ obtained from a second artesian well (Well 2). Both wells were located at the Experimental Farm of CCTA/UFCG, and their physicochemical characteristics are presented in Table 4.

The irrigation water with ECw levels of 1.5 and 2.1 dS m^−1^ was prepared by mixing water from Wells 1 and 2 (Table 4). The ECw levels of 2.7 and 3.3 dS m^−1^ were obtained by dissolving non-iodized NaCl in the water from Well 2 (EC = 2.66 dS m^−1^). The preparation of the saline irrigation water was based on the relationship between irrigation water electrical conductivity and salt concentration, as described by Richards [55], according to Equation (1):

C = 640 × ECw(1)

where

C = Amount of salts to be added (mg L^−1^);

ECw = Desired electrical conductivity of the irrigation water (dS m^−1^), after subtracting the initial electrical conductivity of the source water (2.66 dS m^−1^).

After preparation and adjustment of the target ECw, the irrigation water was stored in 500 L tanks. Irrigation with the different ECw levels was applied daily, with the irrigation volume determined by the soil water balance using Equation (2):

(2) $$\mathrm{IV}=\frac{\mathrm{IVa}-\mathrm{IVb}}{\left(1-\mathrm{LF}\right)}$$

where

IV = Irrigation water volume to be applied at each irrigation event (mL).

IVa = Irrigation water volume applied during the previous irrigation event (mL);

IVd = drained volume (mL);

LF = leaching fraction of 0.15 applied every 15 days.

A drip irrigation system was used, consisting of a 32-mm PVC mainline (PN 40) and 16-mm low-density polyethylene lateral lines. Pressure-compensating emitters (GA 10 Grapa model) with a nominal flow rate of 10 L h^−1^ were installed. Two emitters were positioned per plant, each located 15 cm from the stem.

**Figure 10:**
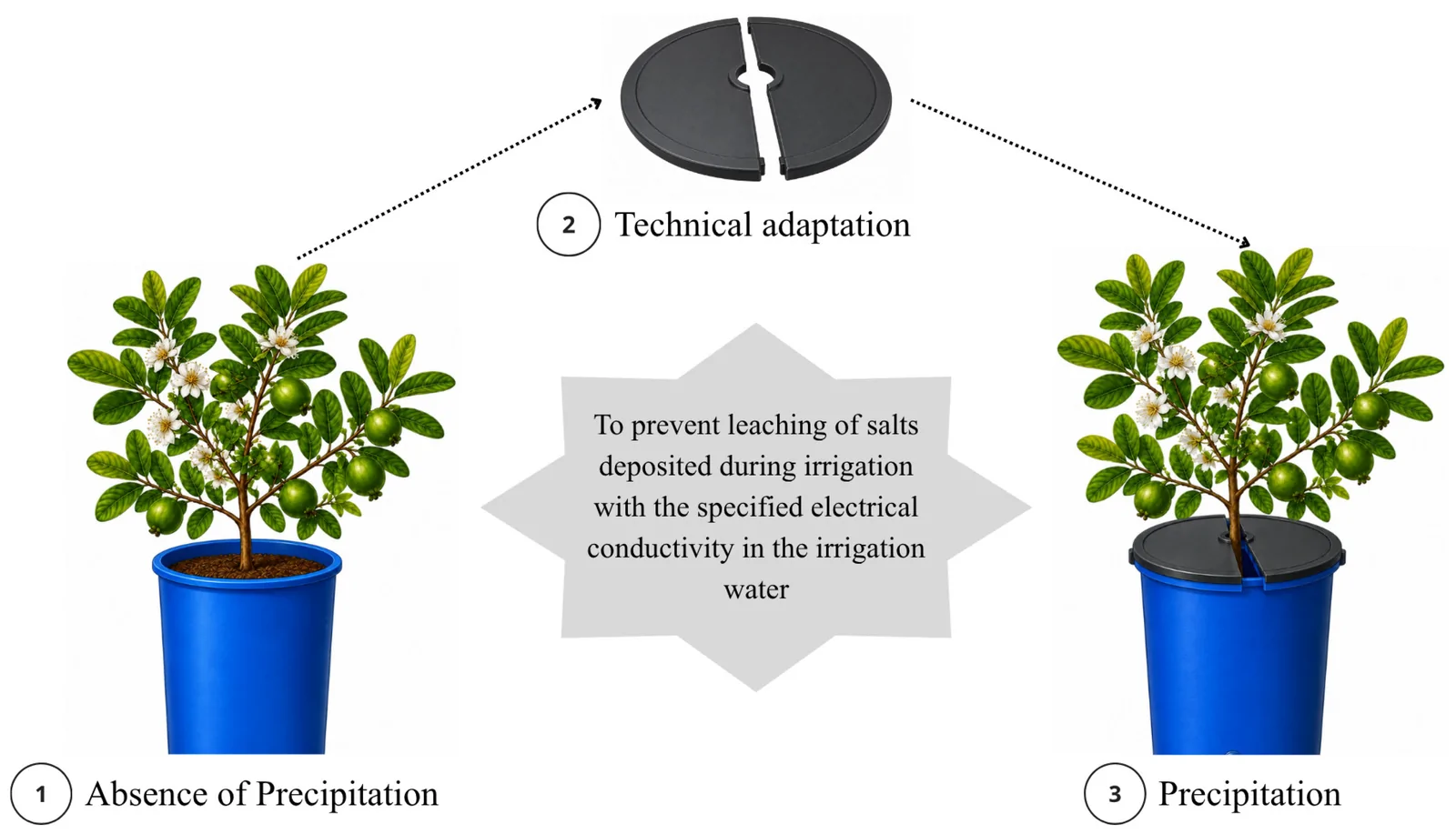
Technical adaptation of the drainage lysimeters to prevent rainfall from interfering with the treatments.

To prevent experimental interference caused by rainfall events during the experimental period, a cover was fitted to the lysimeter (Figure 10) to prevent rainwater from entering and, consequently, avoid changes in the treatments.

### 4.7. Pruning Management

Plants were trained to a single stem until reaching a height of 50 cm. At this stage, the apical bud was removed to stimulate the development of lateral shoots. From the emerging shoots, four well-distributed scaffold branches were selected and oriented toward the four cardinal directions. After the scaffold branches had fully developed, they were pruned to a length of 40 cm to establish the canopy architecture required for fruit production [56].

### 4.8. Preparation and Application of Ascorbic Acid

Ascorbic acid solutions were prepared immediately before each application by dissolving L-ascorbic acid (L-Ascorbic Acid^®^) in tap water. Since the experiment was conducted continuously over two production cycles, from August 2023 to February 2025, foliar Asc application began at 90 days after transplanting (DAT), 72 h before the first irrigation with saline water during the first production cycle, and continued until 210 DAT, when the full flowering period began. Following maintenance pruning at 390 DAT, Asc application was resumed and continued until 480 DAT. Applications were performed at 15-day intervals during both production cycles. To reduce the surface tension of the spray solution and improve leaf coverage, the non-ionic surfactant Tween 20 was added at a concentration of 0.025% (*v*/*v*). Applications were carried out after 17:00 h, and plants were isolated according to their respective treatments using plastic sheeting to prevent spray drift onto adjacent plants. In experimental period, a total of 16 applications were performed, with an average spray solution volume of 727.28 mL plant^−1^ per application, through direct measurement after application to each plant. At each application event, plants in the control treatment (0 mg L^−1^) received only distilled water at the same volume.

### 4.9. Production Components

Fruits were harvested when they reached the commercial ripening stage, characterized by the transition in peel color from green to yellow. After each harvest, fruit polar diameter (FPD), fruit equatorial diameter (FED), average fruit weight (AFW), fruit yield (FY), and the total number of fruits (TNF) were determined.

Fruit polar diameter (FPD) and fruit equatorial diameter (FED) were determined using 10 fruits per plant, randomly selected at each harvest. FPD was measured from the peduncle insertion point to the blossom end of the fruit, whereas FED was measured at the widest portion of the fruit using a digital caliper (Digimess, São Paulo, Brazil). Average fruit weight (AFW) was calculated as the ratio between fruit yield and the total number of harvested fruits. The total number of fruits (TNF) was obtained by direct counting of all harvested fruits, while fruit yield (FY) was determined as the cumulative fresh weight of all fruits harvested from each plant.

### 4.10. Postharvest Fruit Quality

The pulp was manually extracted from the fruits and immediately stored in hermetically sealed containers at −10 °C until analysis. During storage, the samples were protected from light to minimize the potential degradation of biochemical compounds. The fruits were processed using an Arno food processor (model DO1675B2 MP76 Multichef, Arno, Itatiaia, Brazil). After homogenization of the pulp from the 10 fruits, 100 g of pulp was collected and stored in plastic containers for subsequent chemical analyses. All analyses were performed in triplicate. Fruit chemical characterization included determination of pH, total soluble solids (TSS, °Brix), titratable acidity (TA, % citric acid), vitamin C content (Vit. C, mg 100 g^−1^ pulp), maturity index (MI), total sugars (TS, mg 100 g^−1^ pulp), flavonoids (FLA, mg 100 g^−1^ pulp), anthocyanins (ANT, mg 100 g^−1^ pulp), and phenolic content (PC, mg 100 g^−1^ pulp).

Fruit pulp pH was measured using a bench-top pH meter (PHB-550 model, Incoterm, Porto Alegre, Brazil) equipped with a glass membrane electrode and automatic temperature compensation, with a resolution of 0.01 pH units. Total soluble solids (TSS, °Brix) were determined directly from the fruit pulp using a portable refractometer (Brewparts, Indaiatuba, São Paulo, Brazil) with a resolution of 0.2 °Brix, with the sample applied using a disposable dropper pipette (Clauplast, São Paulo, Brazil).

Titratable acidity (TA) was determined according to the methodology described by the Instituto Adolfo Lutz (IAL) [57]. A 3 g pulp sample was weighed into a 125 mL Erlenmeyer flask, followed by the addition of 50 mL of distilled water and two drops of phenolphthalein indicator. The sample was then titrated with 0.1 M sodium hydroxide (NaOH). Titratable acidity, expressed as g citric acid per 100 g of pulp, was calculated according to Equation (3):

(3) TA = g citric acid 100  g=V × f × N ×64.0410× P

where

V = Volume of the NaOH solution (0.1 M) consumed during the titration (in mL);

f = Correction factor for the titrant solution;

N = Normality of the NaOH solution (0.1 M);

64.04 = Conversion factor for citric acid;

P = Mass of the sample used in the analysis (in g).

The maturity index (MI) was calculated as the ratio between total soluble solids (TSS) and titratable acidity (TA). Vitamin C content was determined by titration according to the method described by Benassi and Antunes [58] and Strohecker and Henning [59]. Briefly, a 1 g pulp sample was weighed and mixed with 50 mL of 0.5% oxalic acid solution. The mixture was homogenized using a Vortex mixer (Warmnest^®^, Paris, France) and then titrated with 2,6-dichlorophenolindophenol (DCPIP) solution (factor = 90.0) until the appearance of a persistent light pink endpoint. Vitamin C content was calculated according to Equation (4).

(4) $$\mathrm{Vit}.\;C=(\frac{\mathrm{mg}}{100\;g})(V\;\times \;F\;\times \;100)/\mathrm{Sm}$$

where

V = Volume of DCPIP used in the titration (mL);

F = DCPIP solution factor;

Sm = Sample mass (g).

Total sugars (TS) were determined using the anthrone colorimetric method, according to Yemm and Willis [60], with anhydrous glucose (P.A., 99.5%) as the calibration standard. Quantification was performed using a calibration curve described by the equation (y = 0.1491x + 0.0125) (R^2^ = 0.99). The concentration corresponding to the absorbance of each sample was calculated using the equation (x = (y − 0.0125)/0.1491), where (y) represents absorbance and (x) represents the concentration estimated from the calibration curve. Results were expressed as g 100 g^−1^ pulp.

Flavonoid and anthocyanin contents were determined using the method described by Francis [61]. For extraction, approximately 1.0 g of pulp was homogenized with 10 mL of an extraction solution containing 95% ethanol and 1.5 N hydrochloric acid (HCl) at a ratio of 85:15 (*v*/*v*). The samples were placed in polypropylene tubes protected from light with aluminum foil and kept under refrigeration for 24 h. After this period, the extracts were filtered, and absorbance was measured using a spectrophotometer (K37-UVVIS, Kasvi, Yangzhou, China) at 374 nm for flavonoids and 535 nm for anthocyanins. Flavonoid and anthocyanin contents were calculated using Equation (5). Results were expressed as mg 100 g^−1^ pulp.

(5) $$\mathrm{FLA}\;\mathrm{and}\;\mathrm{ANT}:\;(\frac{\mathrm{mg}}{100\;g})=\frac{\mathrm{DF}\times \mathrm{ABS}}{76.6}$$

where

FD = Dilution factor (2000);

A = Absorbance measured at 374 nm for flavonoids and 535 nm for anthocyanins.

Total phenolic compound content (PC) was determined using the Folin–Ciocalteu method, according to Waterhouse [62]. To prepare the extract, 1.0 g of pulp was homogenized in 50 mL of distilled water and allowed to stand for 30 min. The mixture was then filtered. An aliquot of the filtrate was used in the reaction with the Folin–Ciocalteu reagent, followed by a 5-min resting period. Subsequently, 250 µL of 20% sodium carbonate was added, and the mixture was incubated in a water bath at 40 °C for 30 min. After cooling, absorbance was measured at 765 nm. Quantification was performed using a gallic acid calibration curve, described by the linear regression equation (y = 0.0828x + 0.083) (R^2^ = 0.97). The concentration was calculated according to Equations (6)–(8), and the results were expressed as mg 100 g^−1^ pulp.

(6) $$X=\frac{\mathrm{ABS}-b}{a}$$

(7) $$Z=\frac{P\times \mathrm{Aliquot}}{\mathrm{Dilution}}$$

(8) $$\mathrm{PC}=\frac{\frac{X\times 100}{Z}}{1000}$$

where

ABD = Value observed on the spectrophotometer;

P = Sample weight;

Dilution = 50,000.

### 4.11. Statistical Analysis

The data were initially tested for normality using the Shapiro–Wilk test. Subsequently, the data were subjected to two-factor analysis of variance (ANOVA) in a factorial design in DBC, and treatment effects were evaluated using the F-test (*p* ≤ 0.05). Error I was obtained based on the main plots (irrigation water electrical conductivity levels), whereas Error II was obtained based on the subplots (ascorbic acid concentrations). When significant effects were detected, polynomial regression analysis was performed for irrigation water electrical conductivity (ECw) and ascorbic acid (Asc) concentrations. Significant ECw × Asc interactions were further explored using three-dimensional response surface plots generated with SigmaPlot software (version 12.5). Pearson correlation analysis was then performed among all measured variables at a significance level of *p* ≤ 0.05, using individual plant observations, followed by network analysis to investigate relationships among yield and postharvest quality traits.

The Multi-Trait Genotype–Ideotype Distance Index (MGIDI) was used to rank the combinations of irrigation water electrical conductivity levels and ascorbic acid concentrations based on the combined performance of the evaluated traits [49]. Initially, the data were organized into a treatment × trait matrix and standardized to eliminate the effects of differences in scale among variables. Factor analysis was then performed to group the traits into factors representing distinct dimensions of agronomic performance.

An ideotype was defined according to the desirable response direction of each trait, considering the agronomic objectives of the study. Subsequently, the distance between each treatment and the ideotype was calculated in factor space, and the MGIDI was obtained according to the methodology proposed by Olivoto and Nardino [50]. Treatments with lower MGIDI values were considered closer to the established ideotype, indicating more favorable multi-trait performance across the evaluated traits. In this context, MGIDI was used as a comparative ranking tool for the management treatments.

Pearson correlations were calculated using the cor () function in R. Correlation network analysis was performed using the qgraph () function. Factor analysis was conducted using the factanal () function in R, while the multi-trait genotype–ideotype distance index (MGIDI) was calculated using the mgidi () function from the metan package. All statistical analyses were performed using R software (version 4.1.0), with the metan [63], AgroR [64], ggcorrplot [65], and mclust [66] packages.

## 5. Conclusions

An irrigation water electrical conductivity of 3.3 dS m^−1^ increased soluble solids content in guava fruits, although it reduced several yield components and quality attributes.

Foliar application of ascorbic acid at 600 mg L^−1^ improved fruit development, as evidenced by increases in fruit equatorial and polar diameters, total fruit number, and yield per plant in guava irrigated with water at 0.9 dS m^−1^. At 600 mg L^−1^, ascorbic acid also mitigated the effects of irrigation water electrical conductivity of 3.3 dS m^−1^ on average fruit weight, titratable acidity, vitamin C, and total sugar contents of guava fruits.

According to the analysis based on the Multi-Trait Genotype–Ideotype Distance Index (MGIDI), treatments S1C4, S3C4, and S5C4 showed the greatest proximity to the ideotype, indicating more favorable multi-trait performance than the other combinations of irrigation water electrical conductivity levels and ascorbic acid concentrations.

Further studies should evaluate ascorbic acid concentrations above 600 mg L^−1^, as well as higher salinity levels, to determine the limits of the effectiveness of this strategy and its mitigation capacity under severe stress conditions. Further studies under open-field conditions are also recommended, evaluating different cultivars, rootstocks, and application frequencies across diverse soil and climatic conditions, together with an assessment of the economic feasibility of this technology and the physiological mechanisms underlying guava responses to ascorbic acid application.

## Figures and Tables

**Table 1:** Percentage distribution of the strengths and weaknesses of the selected treatments based on the multi-trait genotype–ideotype distance index (MGIDI), considering the interaction between irrigation water electrical conductivity (ECw) and ascorbic acid (Asc) concentrations, together with the distribution of the principal components (PC1–PC5) and the communality estimates for yield components and fruit physical and chemical attributes.

	Principal Components				
	PC1	PC2	PC3	PC4	PC5
Eigenvalues	4.79	2.81	1.85	1.53	1.02
Variance (%)	34.06	20.10	13.21	10.81	7.25
	Factor analysis—Correlation coefficients (r)				
Variables	FA1	FA2	FA3	FA4	FA5
FDP	−0.47	−0.76 **	0.18	0.1	−0.07
FED	−0.39	−0.32	0.57 **	0.08	0.23
AFW	0.07	−0.37	−0.18	0.89 **	0.04
FY	−0.14	−0.66	−0.17	0.02	0.67 **
TNF	−0.15	−0.28	−0.01	−0.78 **	0.52
pH	0.1	−0.86 **	0.15	0.12	0.25
TSS	0.92 **	−0.15	−0.03	0.2	−0.01
TA	0.31	0.02	−0.88 **	0.2	0.04
Vit C	0.78 **	0.09	−0.06	−0.02	−0.4
MI	0.29	−0.16	0.9 **	−0.1	−0.01
TS	0.23	−0.56	−0.57 **	−0.11	−0.16
FLA	−0.19	−0.1	0.17	−0.18	0.87 **
ANT	−0.35	0.28	0.6 **	0.02	0.5
PC	0.7 **	0.4	−0.36	0.05	−0.19

PC—principal components; **—significant at *p* ≤ 0.01.

**Table 2:** Predicted selection gains for yield components and postharvest quality traits of guava fruits estimated using the Multi-Trait Genotype–Ideotype Distance Index (MGIDI) for the selected treatments.

Variables	Factor	X_0_	Xs	SD	SDperc	h^2^	Sense	Goal
TSS	FA1	13.9	14.7	0.73	5.26	0.82	increase	100
Vit C	FA1	42.3	44.5	2.19	5.18	0.974	increase	100
PC	FA1	56.9	59.2	2.27	3.99	0.966	increase	100
FDP	FA2	58.9	60.9	2.09	3.55	0.935	increase	100
pH	FA2	3.14	3.59	0.46	14.7	0.965	increase	100
FED	FA3	49.9	49.5	−0.37	−0.733	0.873	increase	0
TA	FA3	0.696	0.761	0.066	9.24	0.852	increase	100
MI	FA3	20.3	19.7	−0.66	−3.25	0.794	increase	0
TS	FA3	15.4	15.9	0.504	3.28	0.883	Increase	100
ANT	FA3	0.498	0.506	0.008	1.6	0.885	Increase	100
AFW	FA4	89.8	117	26.80	29.9	0.816	Increase	100
TNF	FA4	17.8	19.9	2.09	11.8	0.866	Increase	100
FY	FA5	1470	1890	420	28.6	0.768	Increase	100
FLA	FA5	10.4	12.9	2.48	23.8	0.981	Increase	100

X_0_—original mean; Xs—mean after selection; SD—selection differential; SDperc—selection differential in percentage (anticipated relevant responses); h^2^—repeatability; Sense—desired direction of selection (increase = increase; decrease = reduce); goal—target value assigned in the ideotype.

**Table 3:** Chemical and physical properties of the soil used in the experiment prior to treatment application.

Chemical Characteristics								
pH H_2_O	OM	P	K^+^	Na^+^	Ca^2+^	Mg^2+^	Al^3+^	H^+^
(1:2.5)	g kg^−1^	(mg kg^−1^)	cmol_c_ kg^−1^					
8.53	3.10	77.30	0.56	0.20	5.08	5.11	0	0
Chemical characteristics				Physical characteristics				
EC_se_	SAR_es_	CEC	TSP	Granulometric fraction			Humidity	
				Sand	Silt	Clay	33.42 kPa^1^	1519.5 kPa^2^
(dS m^−1^)	(mmol L^−1^)^0.5^	cmol_c_ kg^−1^	%	(g kg^−1^)			(dag kg^−1^)	
0.46	1.02	10.95	1.83	775.70	180.90	43.40	12.45	5.00

pH, hydrogen potential; OM, organic matter (Walkley–Black wet oxidation); Ca^2+^ and Mg^2+^, extracted with 1 M KCl (pH 7.0); Na^+^ and K^+^, extracted with 1 M NH_4_OAc (pH 7.0); Al^3+^ + H^+^, extracted with 0.5 M CaOAc (pH 7.0); ECe, electrical conductivity of the saturated soil extract; SAR, sodium adsorption ratio of the saturated soil extract; CEC, cation exchange capacity.

**Table 4:** Chemical characteristics of the water used in the experiment.

Location	Ca^2+^	Mg^2+^	Na^+^	K^+^	SO_4_^2^	HCO_3_^−^	CO_3_^2−^	Cl^−^	ECw (dS m^−1^)	pH	SAR (mmol L^−1^)^0.5^
	(mmol_c_ L^−1^)										
Well 1	1.24	4.66	7.56	0.03	Presence	3.55	0.00	5.70	0.90	7.22	4.40
Well 2	1.53	4.56	30.96	0.03	Presence	5.64	0.00	21.85	2.66	7.34	17.74

EC, electrical conductivity; SAR, sodium adsorption ratio.

## Data Availability

The original contributions presented in this study are included in the article and Supplementary Materials. Further inquiries can be directed to the corresponding author.

## Supplementary Materials

The following supporting information can be downloaded at: https://www.mdpi.com/article/10.3390/plants15213279/s1, Table S1. Summary of the analysis of variance for fruit polar diameter (FPD), fruit equatorial diameter (FED), average fruit weight (AFW), fruit yield (FY), and total number of fruits (TNF) of guava cv. Paluma cultivated under irrigation with saline water and foliar application of ascorbic acid; Table S2. Summary of the analysis of variance for hydrogen potential (pH), total soluble solids (TSS), titratable acidity (TA), vitamin C (Vit. C), and total sugars (TS) of guava cv. Paluma fruits cultivated under irrigation with saline water and foliar application of ascorbic acid; Table S3. Summary of the analysis of variance for maturity index (MI), flavonoids (FLA), anthocyanins (ANT), and phenolic compounds (PC) in the pulp of guava cv. Paluma cultivated under irrigation with saline water and foliar application of ascorbic acid.

## Abbreviations

The following abbreviations are used in this manuscript:

Abbreviation	Variable
FPD	Fruit polar diameter
FED	Fruit equatorial diameter
AFW	Average fruit weight
FY	Fruit yield
TNF	Total number of fruits
pH	Hydrogen potential
TSS	Total soluble solids
TA	Titratable acidity
Vit. C	Vitamin C
TS	Total sugars
MI	Maturity index
FLA	Flavonoids
ANT	Anthocyanins
PC	Phenolic content
S1C1	ECw = 0.9 dS m^−1^ + Asc = 0 mg L^−1^
S1C2	ECw = 0.9 dS m^−1^ + Asc = 200 mg L^−1^
S1C3	ECw = 0.9 dS m^−1^ + Asc = 400 mg L^−1^
S1C4	ECw = 0.9 dS m^−1^ + Asc = 600 mg L^−1^
S2C1	ECw = 1.5 dS m^−1^ + Asc = 0 mg L^−1^
S2C2	ECw = 1.5 dS m^−1^ + Asc = 200 mg L^−1^
S2C3	ECw = 1.5 dS m^−1^ + Asc = 400 mg L^−1^
S2C4	ECw = 1.5 dS m^−1^ + Asc = 600 mg L^−1^
S3C1	ECw = 2.1 dS m^−1^ + Asc = 0 mg L^−1^
S3C2	ECw = 2.1 dS m^−1^ + Asc = 200 mg L^−1^
S3C3	ECw = 2.1 dS m^−1^ + Asc = 400 mg L^−1^
S3C4	ECw = 2.1 dS m^−1^ + Asc = 600 mg L^−1^
S4C1	ECw = 2.7 dS m^−1^ + Asc = 0 mg L^−1^
S4C2	ECw = 2.7 dS m^−1^ + Asc = 200 mg L^−1^
S4C3	ECw = 2.7 dS m^−1^ + Asc = 400 mg L^−1^
S4C4	ECw = 2.7 dS m^−1^ + Asc = 600 mg L^−1^
S5C1	ECw = 3.3 dS m^−1^ + Asc = 0 mg L^−1^
S5C2	ECw = 3.3 dS m^−1^ + Asc = 200 mg L^−1^
S5C3	ECw = 3.3 dS m^−1^ + Asc = 400 mg L^−1^
S5C4	ECw = 3.3 dS m^−1^ + Asc = 600 mg L^−1^
