# Supplementary material for: Ascorbic Acid Promotes Beneficial Effects on Yield Components and Postharvest Quality of Grafted Guava Under Salt Stress
Source: Plants (Basel). 2026 Oct 24;15(21):3279. doi: 10.3390/plants15213279 (PMC13766196; doi:10.3390/plants15213279)
Supplement: Supplementary file 1 [file plants-15-03279-s001.zip › plants-4592550-supplementary.pdf]

Table S1. Summary of the analysis of variance for fruit polar diameter (FPD), fruit equatorial diameter (FED), average fruit weight (AFW), fruit yield per plant (FY), and total number of fruits per plant (TNF) of guava cv. Paluma cultivated under irrigation with saline water and foliar application of ascorbic acid.

Sources of variation	DF	Mean squares				
		FPD	FED	AFW	FY	TNF
Water salinity (ECw)	4	530.56**	158.25**	1952.14*	653646.16*	103.30**
Linear Regression	1	1826.13**	607.00**	1507.97^{ns}	1446322.95**	367.50**
Quadratic Regression	1	94.56*	2.95^{ns}	5683.23**	921301.96*	9.52^{ns}
Residual I - Parcel	8	13.11	13.00	292.06	106413.17	11.17
Ascorbic acid (Asc)	3	122.70**	52.05**	2173.41*	615664.44**	59.43**
Linear Regression	1	256.78**	0.74^{ns}	5358.99*	1598418.23**	25.23^{ns}
Quadratic Regression	1	0.97^{ns}	31.84*	33.52^{ns}	248575.05^{ns}	120.41**
Interaction (ECw × Asc)	12	134.62**	38.33**	4168.92**	338549.84**	84.28**
Blocks	2	0.01*	1.64^{ns}	2209.96*	167086.97^{ns}	10.40^{ns}
Residual II - Subparcel	30	14.20	7.14	709.83	103729.48	11.01
CV 1 (%)		6.15	7.23	19.04	22.19	18.72
CV 2 (%)		6.40	5.36	29.68	21.91	18.59

DF – degrees of freedom; CV (%) – coefficient of variation; ns, **, and * indicate non-significant, significant at $p \leq 0.01$, and significant at $p \leq 0.05$, respectively, by the F-test.

Table S2. Summary of the analysis of variance for hydrogen potential (pH), total soluble solids (TSS), titratable acidity (TA), vitamin C (Vit. C), and total sugars (TS) of guava cv. Paluma fruits cultivated under irrigation with saline water and foliar application of ascorbic acid

Sources of variation	DF	Mean squares				
		pH	TSS	TA	Vit. C	TS
Water salinity (ECw)	4	0.71**	6.20**	0.04**	1077.54**	8.13*
Linear Regression	1	2.02**	17.86**	0.18**	3031.08**	25.26**
Quadratic Regression	1	0.59**	0.05^{ns}	1.00E^{-5ns}	488.03**	0.13^{ns}
Residual I - Parcel	8	0.03	0.21	5.22E^{-3}	7.50	1.94
Ascorbic acid (Asc)	3	8.99**	11.63**	0.01**	451.49**	3.52*
Linear Regression	1	8.30**	33.13**	0.02**	1204.08**	6.78*
Quadratic Regression	1	0.07^{ns}	0.58^{ns}	0.01*	73.04**	3.73^{ns}
Interaction (ECw × Asc)	12	0.43**	1.55^{ns}	0.01**	104.56**	14.66**
Blocks	2	0.12^{ns}	0.73^{ns}	1.71E^{-3ns}	14.00	0.01^{ns}
Residual II - Subparcel	30	0.03	0.88	1.71E^{-3}	9.98	1.19
CV 1 (%)		5.80	3.34	10.39	6.48	9.06
CV 2 (%)		5.60	6.76	7.75	7.47	7.11

DF – degrees of freedom; CV (%) – coefficient of variation; ns, **, and * indicate non-significant, significant at $p \leq 0.01$, and significant at $p \leq 0.05$, respectively, by the F-test.

Table S3. Summary of the analysis of variance for maturity index (MI), flavonoids (FLA), anthocyanins (ANT), and phenolic compounds (PC) in the pulp of guava cv. Paluma cultivated under irrigation with saline water and foliar application of ascorbic acid

Sources of variation	DF	Mean squares			
		MI	FLA	ANT	PC
Water salinity (ECw)	4	24.89*	43.73**	0.02**	4099.55**
Linear Regression	1	56.56**	121.56**	0.08**	15870.69**
Quadratic Regression	1	0.15^{ns}	46.15**	8.86E^{-3ns}	35.41^{ns}
Residual I - Parcel	8	4.14	0.35	2.44E^{-3}	31.90
Ascorbic acid (Asc)	3	9.66^{ns}	22.79**	0.01**	1016.83**

Linear Regression	1	15.27^{ns}	1.33E^{-4ns}	8.86E^{-3**}	145.97^{ns}
Quadratic Regression	1	12.77^{ns}	46.78**	0.01**	464.98**
Interaction (ECw × Asc)	12	21.79**	13.07**	7.76E^{-3**}	438.58**
Blocks	2	0.96^{ns}	0.29^{ns}	8.73E^{-3ns}	26.84^{ns}
Residual II - Subparcel	30	4.24	0.40	1.19E^{-3}	47.09
CV 1 (%)		10.02	5.76	9.91	9.92
CV 2 (%)		10.14	6.10	6.92	12.05

DF – degrees of freedom; CV (%) – coefficient of variation; ns, **, and * indicate non-significant, significant at $p \leq 0.01$, and significant at $p \leq 0.05$, respectively, by the F-test.
